# Urine metabolome profiling of immune-mediated inflammatory diseases

**DOI:** 10.1186/s12916-016-0681-8

**Published:** 2016-09-08

**Authors:** Arnald Alonso, Antonio Julià, Maria Vinaixa, Eugeni Domènech, Antonio Fernández-Nebro, Juan D. Cañete, Carlos Ferrándiz, Jesús Tornero, Javier P. Gisbert, Pilar Nos, Ana Gutiérrez Casbas, Lluís Puig, Isidoro González-Álvaro, José A. Pinto-Tasende, Ricardo Blanco, Miguel A. Rodríguez, Antoni Beltran, Xavier Correig, Sara Marsal, for the IMID Consortium, Emilia Fernández, Raimon Sanmartí, Jordi Gratacós, Víctor Manuel Martínez-Taboada, Fernando Gomollón, Esteban Daudén, Joan Maymó, Rubén Queiró, Francisco Javier López-Longo, Esther Garcia-Planella, José Luís Sánchez-Carazo, Mercedes Alperi-López, Carlos Montilla, José Javier Pérez-Venegas, Benjamín Fernández-Gutiérrez, Juan L. Mendoza, José Luís López-Estebaranz, Àlex Olivé, Juan Carlos Torre-Alonso, Manuel Barreiro-de Acosta, David Moreno-Ramírez, Hèctor Corominas, Santiago Muñoz-Fernández, José Luis Andreu, Fernando Muñoz, Pablo de la Cueva, Alba Erra, Carlos M. González, María Ángeles Aguirre-Zamorano, Maribel Vera, Francisco Vanaclocha, Daniel Roig, Paloma Vela, Cristina Saro, Enrique Herrera, Pedro Zarco, Joan M. Nolla, Maria Esteve, José Luis Marenco de la Fuente, José María Pego-Reigosa, Valle García-Sánchez, Julián Panés, Eduardo Fonseca, Francisco Blanco, Jesús Rodríguez-Moreno, Patricia Carreira, Julio Ramírez, Gabriela Ávila, Laia Codó, Josep Lluís Gelpí, Andrés C. García-Montero, Núria Palau, María López-Lasanta, Raül Tortosa

**Affiliations:** 1 Rheumatology Research Group, Vall d’Hebron Hospital Research Institute, Barcelona, Spain; 2 Centre for Omic Sciences, COS-DEEEA-URV-IISPV, Reus, Spain; 3 Metabolomics Platform, CIBERDEM, Reus, Spain; 4 Hospital Universitari Germans Trias i Pujol, Badalona, Spain; 5 CIBERehd, Madrid, Spain; 6 UGC Reumatología, Instituto de Investigación Biomédica (IBIMA), Hospital Regional Universitario de Málaga, Universidad de Málaga, Málaga, Spain; 7 Hospital Clínic de Barcelona and IDIBAPS, Barcelona, Spain; 8 Hospital Universitario Guadalajara, Guadalajara, Spain; 9 Hospital Universitario de la Princesa and IIS-IP, Madrid, Spain; 10 Hospital la Fe, Valencia, Spain; 11 Hospital General de Alicante, Alicante, Spain; 12 Hospital de la Santa Creu i Sant Pau, Barcelona, Spain; 13 Complejo Hospitalario Juan Canalejo, INIBIC, A Coruña, Spain; 14 Hospital Universitario Marqués de Valdecilla, Santander, Spain

**Keywords:** Metabolomics, Urine biomarkers, Disease activity, Autoimmune diseases, Inflammatory diseases

## Abstract

**Background:**

Immune-mediated inflammatory diseases (IMIDs) are a group of complex and prevalent diseases where disease diagnostic and activity monitoring is highly challenging. The determination of the metabolite profiles of biological samples is becoming a powerful approach to identify new biomarkers of clinical utility. In order to identify new metabolite biomarkers of diagnosis and disease activity, we have performed the first large-scale profiling of the urine metabolome of the six most prevalent IMIDs: rheumatoid arthritis, psoriatic arthritis, psoriasis, systemic lupus erythematosus, Crohn’s disease, and ulcerative colitis.

**Methods:**

Using nuclear magnetic resonance, we analyzed the urine metabolome in a discovery cohort of 1210 patients and 100 controls. Within each IMID, two patient subgroups were recruited representing extreme disease activity (very high vs. very low). Metabolite association analysis with disease diagnosis and disease activity was performed using multivariate linear regression in order to control for the effects of clinical, epidemiological, or technical variability. After multiple test correction, the most significant metabolite biomarkers were validated in an independent cohort of 1200 patients and 200 controls.

**Results:**

In the discovery cohort, we identified 28 significant associations between urine metabolite levels and disease diagnosis and three significant metabolite associations with disease activity (*P*_FDR_ < 0.05). Using the validation cohort, we validated 26 of the diagnostic associations and all three metabolite associations with disease activity (*P*_FDR_ < 0.05). Combining all diagnostic biomarkers using multivariate classifiers we obtained a good disease prediction accuracy in all IMIDs and particularly high in inflammatory bowel diseases. Several of the associated metabolites were found to be commonly altered in multiple IMIDs, some of which can be considered as hub biomarkers. The analysis of the metabolic reactions connecting the IMID-associated metabolites showed an over-representation of citric acid cycle, phenylalanine, and glycine-serine metabolism pathways.

**Conclusions:**

This study shows that urine is a source of biomarkers of clinical utility in IMIDs. We have found that IMIDs show similar metabolic changes, particularly between clinically similar diseases and we have found, for the first time, the presence of hub metabolites. These findings represent an important step in the development of more efficient and less invasive diagnostic and disease monitoring methods in IMIDs.

**Electronic supplementary material:**

The online version of this article (doi:10.1186/s12916-016-0681-8) contains supplementary material, which is available to authorized users.

## Background

Rheumatoid arthritis (RA), psoriasis (Ps), psoriatic arthritis (PsA), systemic lupus erythematosus (SLE), Crohn’s disease (CD), and ulcerative colitis (UC) are prevalent immune-mediated inflammatory diseases (IMIDs) [1–4]. This group of diseases is characterized by the aberrant and chronic activation of the immune system, affecting one or more tissues. IMIDs have a high socioeconomic impact [1, 4, 5] and are among the main causes of morbidity, disability, and mortality in developed countries [6–8]. Although each IMID targets different tissues and organs, they all share common molecular mechanisms like the activation of the Tumor Necrosis Factor cytokine pathway [9]. Recently, genome-wide association studies have demonstrated that IMIDs also share many genetic risk loci [10]. Consequently, the combined analysis of multiple IMIDs has the ability to leverage the identification of more relevant molecular features.

Improvements in the diagnosis of IMIDs would be of great benefit to the patient and would significantly reduce the socioeconomic burden of these diseases. There is increasing evidence that the administration of therapies, particularly biological treatments, at earlier stages of the disease results in a more effective control of the inflammatory process [11, 12]. In RA, for example, early diagnosis and treatment have been shown to increase the probability of entering disease remission [13–16], an accomplishment that was unthinkable only a decade ago. Similarly, the diagnosis of inflammatory bowel diseases CD and UC is often established too late, when severe complications have already occurred [17]. The identification of more accurate diagnostic biomarkers would therefore have a high impact on the improvement of disease outcomes in IMIDs.

Measuring disease activity is also a challenging problem in IMIDs. The lack of objective and highly informative markers of disease activity has a negative impact in key aspects of patient management, like the decision to initiate or terminate a specific therapy. Currently, different scores are available to measure disease activity in each IMID. These scores are based on clinical, laboratory, and/or imaging measures, and although they are frequently used in clinical practice, they have important limitations [18]. Disease activity scores are often based on unspecific and sometimes subjective variables that significantly increase their inter- and intra-observer variability, clearly reducing their accuracy and, consequently, affecting disease monitoring [19]. The dynamic nature and highly informative properties of biological molecules (i.e., biomarkers) could provide the level of objectivity and accuracy necessary for a better management of disease activity in IMIDs.

High-throughput analysis technologies are able to generate comprehensive profiles of different molecular species from multiple biological samples. Recent developments in these technologies could provide the level of precision that is required to improve disease management [20–22]. However, one limitation in the use of these approaches to study IMIDs is that the target tissue or organ cannot be easily sampled, resulting in a highly invasive procedure. Instead, the use of more accessible surrogate tissues or biofluids like blood, saliva and urine could help to circumvent this limitation. Urine, in particular, is a highly interesting sample source since its collection is very simple and is clearly non-invasive for the patient. The direct relationship with blood composition strongly supports the hypothesis that different molecular species that are present in both biological fluids like metabolites, nucleic acids, or proteins and whose variation is associated with pathological features could be highly informative biomarkers in IMIDs [23, 24].

The profiling of the metabolite composition of biological samples, metabolomics, is one of the most rapidly evolving high-throughput analysis approaches [25]. Metabolites could potentially serve as biomarkers in many diseases since they represent the biochemical end products of the genetic pathways, providing an accurate representation of the physiological state of an individual [26]. Nuclear magnetic resonance (NMR), together with mass spectrometry, is one of the most widely used metabolomic technologies [27]. NMR has been used in the determination of the metabolite profiles of tissue and biofluid samples of multiple diseases [28, 29]. To date, however, very few studies have analyzed the metabolomic profiles of IMIDs and most lack independent validation cohorts. Further, there is a lack of studies comparing the metabolomes of this group of inflammatory diseases in parallel.

In the present work, we have performed a large-scale high-throughput analysis of the urine metabolome of six of the most prevalent IMIDs (RA, PsA, Ps, SLE, CD, and UC) and a cohort of healthy control individuals in order to identify new biomarkers associated with disease diagnosis and disease activity. For this objective, we have used a two-stage study design consisting of a discovery stage where the urine metabolomes of 1210 IMID patients and 100 healthy controls were analyzed, and a validation stage where the most significant candidate metabolite biomarkers from the discovery stage were confirmed using an independent cohort of 1200 IMID patients and 200 healthy controls. To our knowledge, this study provides the first comprehensive characterization of urine metabolites associated with IMIDs.

## Methods

### Study design

A two-stage approach was used to characterize the urine metabolite profile associated with IMIDs. In the first stage (discovery stage), candidate biomarkers for diagnosis and disease activity monitoring were identified using a cohort of 1310 individuals (*n* = 1210 IMID patients and *n* = 100 healthy controls). In the second stage (validation stage), the most significant candidate biomarkers where validated using a cohort of 1400 individuals (*n* = 1200 IMID patients and *n* = 200 healthy controls). In order to identify urine metabolites associated with disease activity, two similarly sized subgroups of patients showing extreme disease activity (i.e., very high and very low disease activity) were selected within each IMID disease (Table 1, Additional file 1: Figure S1). Previous metabolomic studies have shown that several epidemiological and technical variables can act as confounders and, therefore, particular care must be taken to avoid or minimize their effects. In the present study, two different measures were taken to reduce the impact of potential confounders. First, the patients and controls from the discovery and validation stages were selected so that they had similar distributions of epidemiological (gender, age and body mass index) and sample collection variables (fasting time of the individual before sample collection and the time of the day of sample collection). Second, in order to adjust for any additional confounding effect, all potential confounder variables were also included as covariates in the multivariate linear regression models testing for association with disease and with disease activity.

**Table 1:** Distribution of sample size and disease activity scores in the low and high activity groups of each immune-mediated inflammatory disease (IMID) after quality control

IMID	Disease activity score	Discovery cohort		Validation cohort	
		Low activity^a^	High activity^a^	Low activity^a^	High activity^a^
CD	Harvey-Bradshaw Index [71]	154 (0.0)	45 (7.0)	100 (0.0)	100 (9.0)
UC	Lichtiger Score [72]	124 (0.0)	81 (6.0)	99 (0.0)	98 (5.0)
RA	Disease Activity Score 28 [73]	114 (1.7)	127 (5.5)	98 (1.6)	95 (5.6)
PsA	Disease Activity Score 28 [73]	97 (1.5)	89 (3.9)	96 (1.5)	96 (4.2)
Ps	Psoriasis Area Severity Index [74]	101 (0.0)	84 (14.1)	100 (0.0)	92 (17.5)
SLE	Selena-Sledai [75] and BILAG [76]	123 (1.0^b^)	41 (11.0^b^)	90 (1.0^b^)	88 (7.5^b^)

Description of the sample sizes and median disease activity values in the discovery and validation stages for each IMID (CD, Crohn’s disease; UC, ulcerative colitis; RA, rheumatoid arthritis; PsA, psoriatic arthritis; Ps, psoriasis; SLE, systemic lupus erythematosus)

^a^Number of samples (median disease activity values)

^b^Maximum of Selena-Sledai and BILAG indices of each patient

### Ethics

The study was conducted according to the Declaration of Helsinki. Patients and controls included in the analysis were recruited by the Immune-Mediated Disease Consortium [29–32]. Informed consent was obtained from all participants, and protocols were reviewed and approved by local institutional review boards. All the patients included in the study met the corresponding consensus diagnostic criteria of each IMID (Additional file 1: Supplementary Methods).

### Metabolomic analysis

Urine samples were collected, processed, and analyzed using ^1^H-NMR as described in the Supplementary Methods (Additional file 1). Spectral processing of the urine NMR profiles was performed using FOCUS software [33], and reference metabolite databases [34] were used to identify the molecules corresponding to each spectral resonance. In order to confirm the identity of specific metabolites, two-dimensional ^1^H-^13^CHSQC (heteronuclear single quantum correlation) and ^1^H-^1^H COSY (correlation spectroscopy) was used in a selected group of samples.

### Statistical analysis

Multivariate linear regression was carried out to test the association between metabolite levels and disease diagnosis as well as disease activity [35–37]. In each linear regression analysis, different epidemiological (i.e., sex, age, smoking habit, body mass index, lifestyle, and dietary habits) and technical variables (i.e., time at sample collection and fasting time) were included as covariates in order to control for confounding. To avoid the presence of false positives associated to drug treatment, we also tested the association between all metabolite levels and drug treatment at the time of sample collection. The drug treatments tested for association included antibody to tumor necrosis factor (anti-TNFα) therapy (i.e., infliximab and etanercept), disease-modifying drugs (i.e., methotrexate and leflunomide), corticoids, and non-steroidal anti-inflammatory drugs (i.e., ibuprofen). After removing known drug-specific metabolites (i.e., ibuprofen, acetaminophen, and 5-aminosalicylic acid) we found no significant association between urine metabolite levels and the presence of any particular therapy.

In the discovery phase, three types of analyses were performed: (1) diagnostic, comparing the metabolite levels between each IMID disease against the healthy control cohort, (2) differential, comparing the metabolite levels between IMIDs that have more similar clinical features, and (3) activity-related, comparing the metabolite concentrations between patients with high and low disease activity within each IMID. Multiple test correction of the significance *P* values was performed using the discovery rate method (false discovery rate (FDR) < 0.05) both in the discovery and validation stages. The hierarchical clustering of urine IMID profiles was performed using the combined association (–log_10_*P* values) for each disease obtained in the case-control analysis.

In order to evaluate the power of the urine metabolome for disease diagnosis, we built a classifier for each IMID using the partial least squares discriminant analysis method in the discovery dataset as described previously [38]. Once the optimal classifier was identified, it was subsequently tested using the independent validation dataset. The performance of the different disease classifiers was determined using the receiver operating characteristic (ROC) curve analysis as described previously [23, 38]. From each ROC, the area under the curve (AUC) statistic was estimated as a measure of the classifier’s diagnostic performance.

In order to gain further biological insight of the associated metabolites, we used the MetaboNetworks software [39]. This method uses a set of predefined metabolic reactions in a single or multiple organisms to identify and define the shortest metabolic reaction chains linking a set of input metabolites. Here, we applied this network analysis approach to identify the shortest metabolic reaction chains linking all metabolites significantly associated with one or more IMIDs. For this analysis we used the set of KEGG reactions (Kyoto Encyclopedia of Genes and Genomes [40]) described for humans as well as the pathways associated with the most abundant endosymbionts from the gut microbiota (Firmicutes, Bacteroidetes, Alphaproteobacteria, Betaproteobacteria, Deltaproteobacteria, Gammaproteobacteria, and Actinobacteria phyla [41]).

## Results

### Sample characteristics and quality control

In the discovery dataset, 1210 IMID patients (203 CD, 213 UC, 250 RA, 167 SLE, 190 PsA, and 187 Ps) and 100 healthy subjects were included in the study. After quality control analysis of the resulting NMR urine spectra, the final discovery dataset consisted of 1180 IMID patient samples and 93 healthy control samples (Additional file 1: Supplementary Methods, Table S1).

The validation dataset used consisted of 1200 IMID patients (*n* = 200 patients per disease) and 200 healthy control subjects. After the quality control analysis of the urine NMR spectra, the final validation dataset consisted of 1152 patient and 196 control samples (Additional file 1: Table S1).

Within each IMID, patients were selected to represent two similarly sized groups of extreme disease activity (i.e., very low and very high disease activity). The average disease activity values for each subgroup are shown in Table 1 and Figure S1 (Additional file 1). The main clinical and epidemiological characteristics of the two cohorts as well as technical variables associated with the sample collection process are presented in Figure S2 (Additional file 1).

### Metabolite panel

A total of 143 spectral peaks were identified in the urine NMR spectra from the discovery dataset. After quality control analysis and filtering of redundant peaks (i.e. peaks quantifying thee same metabolite), a final set of *n* = 37 unique metabolites was identified. To improve this metabolite identification stage, two-dimensional ^1^H-^13^CHSQC and ^1^H-^1^H COSY were performed to validate and resolve unclear metabolite assignments. From these, 37 metabolites identified, of which four metabolites (ibuprofen, acetaminophen, 5-aminosalicylic acid, and ethanol) were found to be either exogenous or drug-related molecules and were excluded from downstream analyses. From the final set of 33 urine metabolites, 25 could be confidently assigned to a known molecule, while the remaining 8 metabolites could not be associated to a known small molecule and therefore were defined using the prefix *Uknown* (Additional file 1: Table S2). According to the Human Metabolome Database [34] all the known metabolites are expected to be found in human urine, and most of them (*n* = 23, > 90 %) have been previously measured in human urine using NMR [42–44].

### Assessment of urine diagnostic biomarkers for IMIDs

**Fig. 1:**
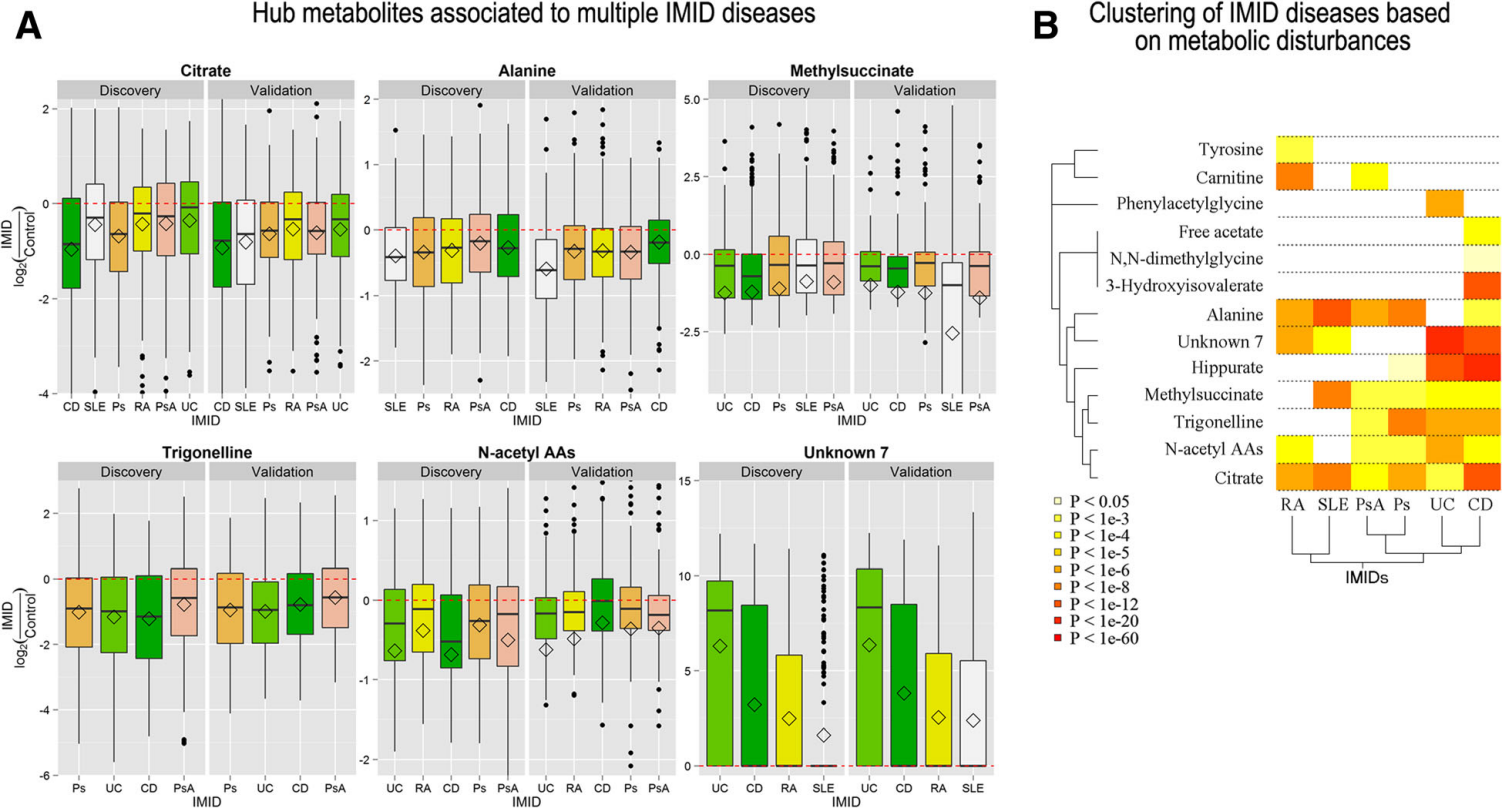
Metabolic reaction network illustrating metabolic signatures associated to IMIDs. Red-shaded metabolites have been associated to IMIDs in the current study. The associated IMIDs are displayed in a text box next to the corresponding metabolite. Disease associations meeting multiple test correction (FDR < 0.05) at the discovery and validation stages are displayed in green letters. Nominal disease associations (*P* < 0.05) at the discovery and validation stages are displayed in red letters. The metabolite reaction linking hippurate and glycine is only conducted through the activity of the gut microbiota

In the discovery stage, the comparison between the urine metabolite profiles between patients and controls identified a total of 28 significant associations (FDR < 0.05). In the validation stage, *n* = 26 of these metabolite associations (93 %) were significantly replicated (FDR < 0.05, Table 2). In a secondary analysis, we found *n* = 13 metabolite associations to be significant at the nominal level in both stages of study (*P* < 0.05, same direction of change, Table 2). Using MetaboNetworks to analyze the associated metabolite profiles [39] we found a overrepresentation of metabolites from the citric acid cycle, phenylalanine metabolism and glycine-serine metabolism pathways (Fig. 1).

**Table 2:** Metabolites associated with each immune-mediated inflammatory disease (IMID) in the discovery and validation cohorts

IMID/Metabolite	log_2_(IMID/Ctrl)_DIS_^b^	*P*_*DIS*_	log_2_(IMID/Ctrl)_VAL_^b^	*P*_*VAL*_	*P*_*COMB*_
CD/Citrate	–0.94 (–1.21 to –0.68)	1.4 × 10^–8^	–0.80 (–1.01 to –0.59)	1.1 × 10^–9^	6.2 × 10^–16^
SLE/Citrate	–0.68 (–0.96 to –0.41)	6.1 × 10^–5^	–0.85 (–1.11 to –0.59)	1.4 × 10^–7^	2.3 × 10^–10^
Ps/Citrate	–0.60 (–0.82 to –0.38)	1.0 × 10^–5^	–0.45 (–0.64 to –0.26)	1.3 × 10^–4^	2.9 × 10^–8^
RA/Citrate	–0.49 (–0.72 to –0.26)	5.4 × 10^–4^	–0.55 (–0.77 to –0.33)	4.3 × 10^–5^	4.3 × 10^–7^
PsA/Citrate	–0.39 (–0.63 to –0.16)	6.7 × 10^–3^	–0.44 (–0.64 to –0.24)	3.7 × 10^–4^	3.5 × 10^–5^
UC/Citrate^a^	–0.34 (–0.57 to –0.11)	1.6 × 10^–2^	–0.39 (–0.59 to –0.19)	1.6 × 10^–3^	3.0 × 10^–4^
UC/N-acetyl AAs	–0.57 (–0.85 to –0.30)	7.6 × 10^–4^	–0.64 (–0.87 to –0.42)	2.4 × 10^–6^	3.8 × 10^–8^
RA/N-acetyl AAs^a^	–0.32 (–0.53 to –0.10)	1.7 × 10^–2^	–0.61 (–0.86 to –0.37)	4.3 × 10^–5^	1.1 × 10^–5^
CD/N-acetyl AAs	–0.63 (–0.90 to –0.36)	1.4 × 10^–4^	–0.26 (–0.47 to –0.06)	3.7 × 10^–2^	6.7 × 10^–5^
Ps/N-acetyl AAs	–0.27 (–0.43 to –0.11)	6.9 × 10^–3^	–0.35 (–0.54 to –0.15)	3.3 × 10^–3^	2.6 × 10^–4^
PsA/N-acetyl AAs	–0.43 (–0.67 to –0.19)	3.9 × 10^–3^	–0.28 (–0.46 to –0.10)	9.1 × 10^–3^	3.9 × 10^–4^
Ps/Trigonelline	–0.70 (–0.99 to –0.40)	1.0 × 10^–4^	–0.73 (–0.94 to –0.51)	7.3 × 10^–8^	2.0 × 10^–10^
UC/Trigonelline	–0.56 (–0.88 to –0.25)	3.3 × 10^–3^	–0.72 (–0.95 to –0.48)	7.3 × 10^–7^	5.0 × 10^–8^
CD/Trigonelline	–0.71 (–1.01 to –0.41)	1.3 × 10^–4^	–0.54 (–0.75 to –0.32)	3.8 × 10^–5^	1.0 × 10^–7^
PsA/Trigonelline^a^	–0.46 (–0.74 to –0.18)	7.5 × 10^–3^	–0.42 (–0.63 to –0.21)	1.1 × 10^–3^	1.0 × 10^–4^
SLE/Alanine	–0.29 (–0.44 to –0.14)	1.5 × 10^–3^	–0.62 (–0.75 to –0.49)	4.8 × 10^–14^	2.7 × 10^–15^
Ps/Alanine	–0.31 (–0.45 to –0.16)	4.2 × 10^–4^	–0.35 (–0.46 to –0.25)	6.5 × 10^–8^	7.0 × 10^–10^
PsA/Alanine^a^	–0.17 (–0.31 to –0.04)	4.0 × 10^–2^	–0.37 (–0.47 to –0.26)	4.0 × 10^–8^	3.4 × 10^–8^
RA/Alanine	–0.24 (–0.38 to –0.10)	5.3 × 10^–3^	–0.35 (–0.46 to –0.24)	4.4 × 10^–7^	4.9 × 10^–8^
CD/Alanine^a^	–0.23 (–0.38 to –0.08)	1.2 × 10^–2^	–0.21 (–0.32 to –0.11)	6.7 × 10^–4^	1.1 × 10^–4^
SLE/Methylsuccinate^a^	–0.72 (–1.25 to –0.19)	2.5 × 10^–2^	–2.10 (–2.61 to –1.59)	5.0 × 10^–11^	3.6 × 10^–11^
UC/Methylsuccinate	–1.09 (–1.58 to –0.60)	2.9 × 10^–4^	–0.61 (–1.01 to –0.22)	1.1 × 10^–2^	4.5 × 10^–5^
CD/Methylsuccinate	–1.05 (–1.56 to –0.54)	7.4 × 10^–4^	–0.67 (–1.08 to –0.26)	7.6 × 10^–3^	7.4 × 10^–5^
PsA/Methylsuccinate^a^	–0.74 (–1.25 to –0.23)	1.6 × 10^–2^	–0.91 (–1.35 to –0.47)	6.7 × 10^–4^	1.4 × 10^–4^
Ps/Methylsuccinate	–0.95 (–1.49 to –0.40)	4.7 × 10^–3^	–0.63 (–1.07 to –0.20)	1.7 × 10^–2^	8.3 × 10^–4^
UC/*Unknown 7*	5.49 (4.72 to 6.26)	2.2 × 10^–26^	5.30 (4.68 to 5.93)	1.5 × 10^–36^	4.7 × 10^–60^
CD/*Unknown 7*	2.40 (1.61 to 3.20)	1.0 × 10^–6^	2.75 (2.14 to 3.36)	5.1 × 10^–13^	2.3 × 10^–17^
RA/*Unknown 7*	1.68 (0.99 to 2.37)	7.6 × 10^–5^	1.48 (0.94 to 2.02)	8.2 × 10^–6^	1.4 × 10^–8^
SLE/*Unknown 7* ^a^	0.81 (0.18 to 1.44)	3.4 × 10^–2^	1.33 (0.80 to 1.86)	4.8 × 10^–5^	2.3 × 10^–5^
CD/Hippurate	–1.74 (–2.08 to –1.40)	1.5 × 10^–15^	–1.54 (–1.80 to –1.28)	4.5 × 10^–20^	5.5 × 10^–33^
UC/Hippurate	–1.03 (–1.32 to –0.73)	3.3 × 10^–8^	–0.92 (–1.16 to –0.69)	3.8 × 10^–10^	4.9 × 10^–16^
Ps/Hippurate^a^	–0.40 (–0.66 to –0.13)	1.6 × 10^–2^	–0.33 (–0.54 to –0.11)	1.4 × 10^–2^	2.0 × 10^–3^
RA/Carnitine^a^	–0.68 (–1.11 to –0.25)	9.2 × 10^–3^	–1.05 (–1.35 to –0.75)	2.1 × 10^–8^	4.4 × 10^–9^
PsA/Carnitine^a^	–0.55 (–0.98 to –0.11)	3.9 × 10^–2^	–0.80 (–1.11 to –0.50)	2.0 × 10^–5^	1.2 × 10^–5^
CD/3-Hydroxyisovalerate	–1.02 (–1.51 to –0.53)	6.7 × 10^–4^	–1.88 (–2.27 to –1.48)	4.2 × 10^–14^	1.1 × 10^–15^
UC/Phenylacetylglycine	0.42 (0.19 to 0.65)	2.5 × 10^–3^	0.48 (0.31 to 0.65)	5.5 × 10^–6^	2.7 × 10^–7^
CD/Free acetate^a^	–0.46 (–0.80 to –0.11)	3.0 × 10^–2^	–0.51 (–0.71 to –0.30)	6.5 × 10^–5^	2.8 × 10^–5^
RA/Tyrosine	1.25 (0.60 to 1.90)	1.7 × 10^–3^	0.57 (0.14 to 1.00)	3.0 × 10^–2^	5.7 × 10^–4^
CD/N,N-dimethylglycine^a^	–0.27 (–0.46 to –0.07)	2.3 × 10^–2^	–0.25 (–0.44 to –0.06)	2.8 × 10^–2^	5.5 × 10^–3^

Association statistics for the discovery (DIS), the validation (VAL) and the combined (COMB) cohorts. ^a^Nominal association

^b^Logarithmic concentration ratios (median, 95 % CI) of the corresponding IMID cohort versus the control cohort

CD, Crohn’s disease; N-acetyl AAs, N-acetyl amino acids; Ps, psoriasis; PsA, psoriatic arthritis; RA, rheumatoid arthritis; SLE, systemic lupus erythematosus; UC, ulcerative colitis

**Fig. 2:**
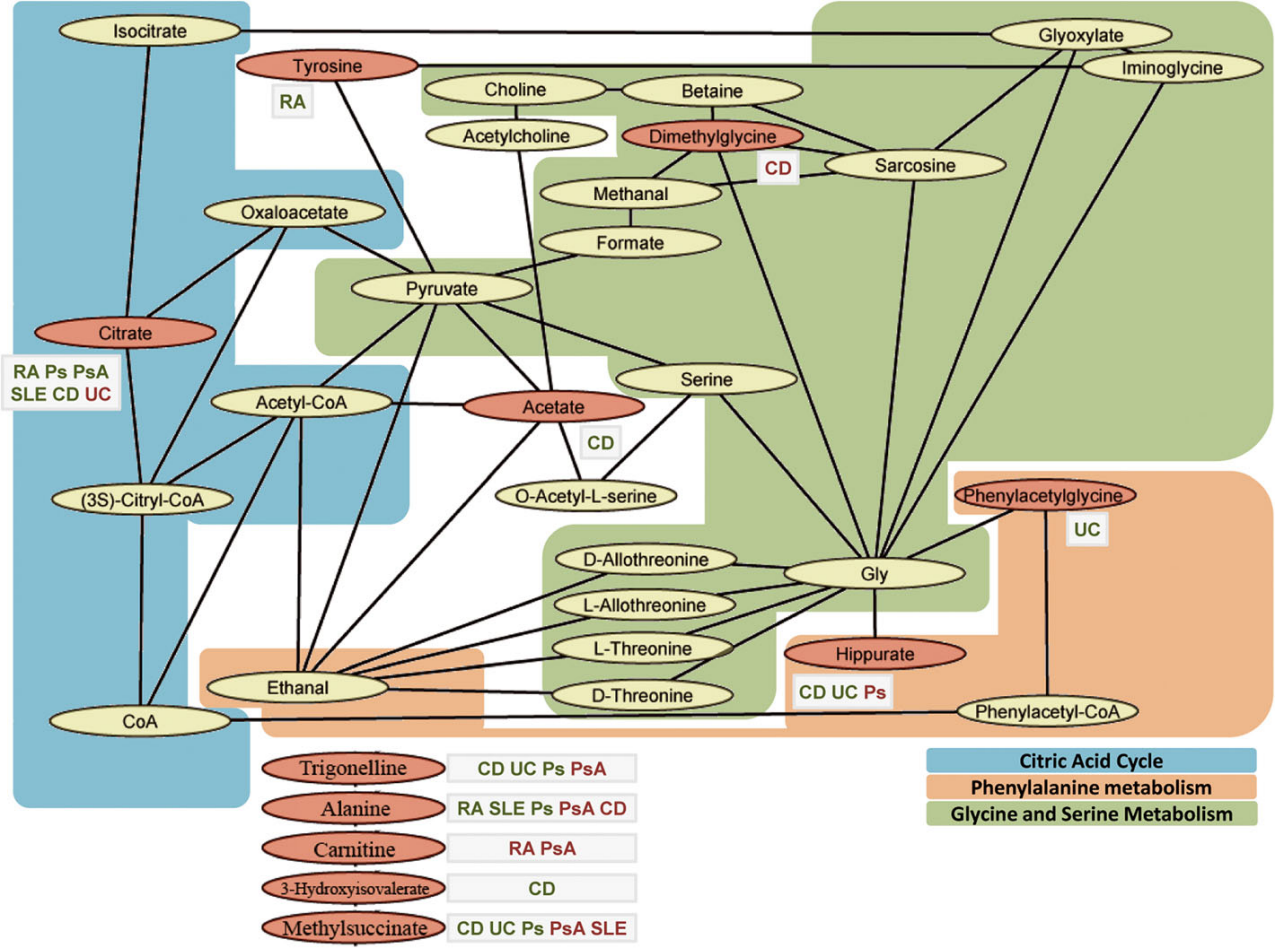
Urine diagnostic biomarkers in IMID diseases. **a** Shows the distribution of the concentrations in logarithmic scale of the metabolites associated to multiple IMID diseases (i.e., hub metabolites). The concentrations have been previously normalized to the median concentration of the control cohort. **b** Shows the clustering graph of both diseases and metabolites according to their corresponding disease-metabolite associations

Among the validated metabolites, six were found to be associated to three or more IMIDs (Fig. 2a). Since their patterns were very similar between diseases (i.e., significance of association and direction of change), they were considered as hub metabolites in IMIDs. From these, citrate showed the strongest hub properties, showing a significantly lower concentration in the urine of most IMIDs compared to controls (Fig. 2, *P*_CD_ = 6.2 × 10^–16^, *P*_SLE_ = 2.3 × 10^–10^, *P*_Ps_ = 2.9 × 10^–8^, *P*_RA_ = 4.3 × 10^–7^, *P*_PsA_ = 3.5 × 10^–5^). In UC, citrate levels were also lower than in controls both in the discovery and validation cohorts, although the difference was only significant at the nominal (*P* < 0.05) level.

Similarly, five other hub metabolites were found to be significantly associated to multiple IMIDs. N-acetyl amino acids (N-acetyl AAs), alanine, methylsuccinate, and trigonelline showed lower concentrations in the urine of several different IMIDs compared to healthy normal controls (Table 2). From these, trigonelline has been previously shown to be associated to the consumption of coffee and tea. Our analysis shows that this metabolite remains significantly associated with different IMIDs even after adjusting for the daily consumption of coffee and/or tea, thereby discarding the possibility of a diet-based confounding (*P* = 4.2 × 10^–6^ and r^2^ = 0.47 in the discovery cohort; Additional file 1: Figures S3 and S4). In addition to these metabolites, urine metabolite *Unknown 7* was found to be present at high levels in the urine metabolome of CD, UC, and RA patients compared to controls (Table 2).

A group of metabolites were found to have differential levels in urine only in IMIDs, with a more similar clinical phenotype. Hippurate levels were found to be significantly lower in the two inflammatory bowel diseases CD and UC compared to controls (Table 2). In the two chronic arthritis diseases, RA and PsA, low levels of carnitine were identified in the discovery stage and replicated in the validation stage (Table 2).

Finally, five metabolites were found to have a differential urine concentration in only one IMID. These disease-specific metabolites include phenylacetylglycine in UC (*P*_UC_ = 2.7 × 10^–7^), tyrosine in RA (*P*_RA_ = 5.7 × 10^–4^), and 3-hydroxyisovaleric (*P*_CD_ = 1.1 × 10^–15^), free acetate (*P*_CD_ = 2.8 × 10^–5^), and N,N-dimethylglycine in CD (*P*_CD_ = 5.5 × 10^–3^) (Table 2).

In order to assess the similarities between the urine metabolic profiles of the different IMIDs, we performed a clustering analysis (Fig. 2b). This analysis showed that the urine metabolite profiles of IMIDs aggregate into three main clusters: (1) Ps and PsA (sharing *n* = 5 metabolite associations), (2) CD and UC (sharing *n* = 6 metabolite associations), and (3) RA and SLE (sharing *n* = 3 metabolite associations).

### Urine metabolomic classifier for IMID diagnosis

**Fig. 3:**
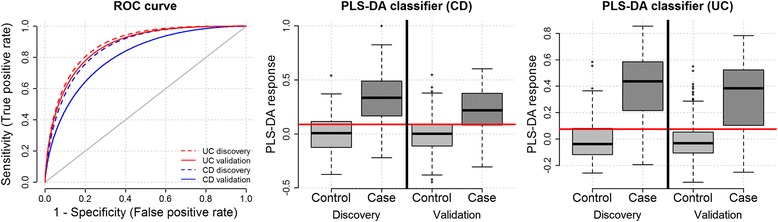
Performance of diagnostic classification models for inflammatory bowel diseases. Distribution of the partial least squares discriminant analysis response variable in the discovery and validation datasets using the same model. The red line shows the optimal classification threshold computed within the discovery cohort

In order to evaluate the power of the urine metabolome for disease diagnosis, a multivariate classification model was built for each IMID disease using the discovery cohort. In order to obtain an independent and non-biased assessment of the diagnostic accuracy of the metabolomic classifiers, these were tested in the validation cohort. Using this approach, the prediction accuracy was found to be high for SLE (AUC_SLE_ = 0.73, 95 % CI, 0.68–0.78), RA (AUC_RA_ = 0.70, 95 % CI, 0.65–0.75), Ps (AUC_PS_ = 0.70, 95 % CI, 0.64–0.75), and PsA (AUC_PSA_ = 0.69, 95 % CI, 0.63–0.74). The metabolomic classifiers from the two bowel inflammatory diseases, CD and UC, showed the strongest diagnostic performance (Fig. 3, Additional file 1: Figure S5). Using the metabolite levels in urine, both CD and UC could be predicted with an AUC higher than 0.80 (AUC_UC_ = 0.87, 95 % CI, 0.83–0.91 and AUC_CD_ = 0.81, 95 % CI, 0.76–0.86).

### Urine biomarkers for differential diagnosis in IMIDs

The metabolite profiles of IMIDs showing a more similar clinical phenotype were directly compared, i.e., CD versus UC, RA versus PsA, Ps versus PsA, and RA versus SLE. In the discovery dataset, a total of 11 metabolites were found to be significantly different between similar IMIDs (FDR < 0.05, Additional file 1: Table S3). From these, three metabolite associations were replicated in the validation cohort (FDR < 0.05, Additional file 1: Table S3). These three validated differential diagnostic metabolites were all found when comparing the profiles of the two inflammatory bowel diseases UC and CD: hippurate (*P* = 9.2 × 10^–8^), citrate (*P* = 1.6 × 10^–8^), and *Unknown 7* (*P* = 6.7 × 10^–18^). All three metabolites showed lower concentrations in the urine of CD patients compared to the urine of UC patients. At the nominal level, tyrosine amino acid (*P* = 1.8 × 10^–4^) and *Unknown 7* metabolite (*P* = 7.9 × 10^–5^) were also found to be lower in the urine of PsA patients compared to RA patients.

### Urine biomarkers of disease activity in IMIDs

**Fig. 4:**
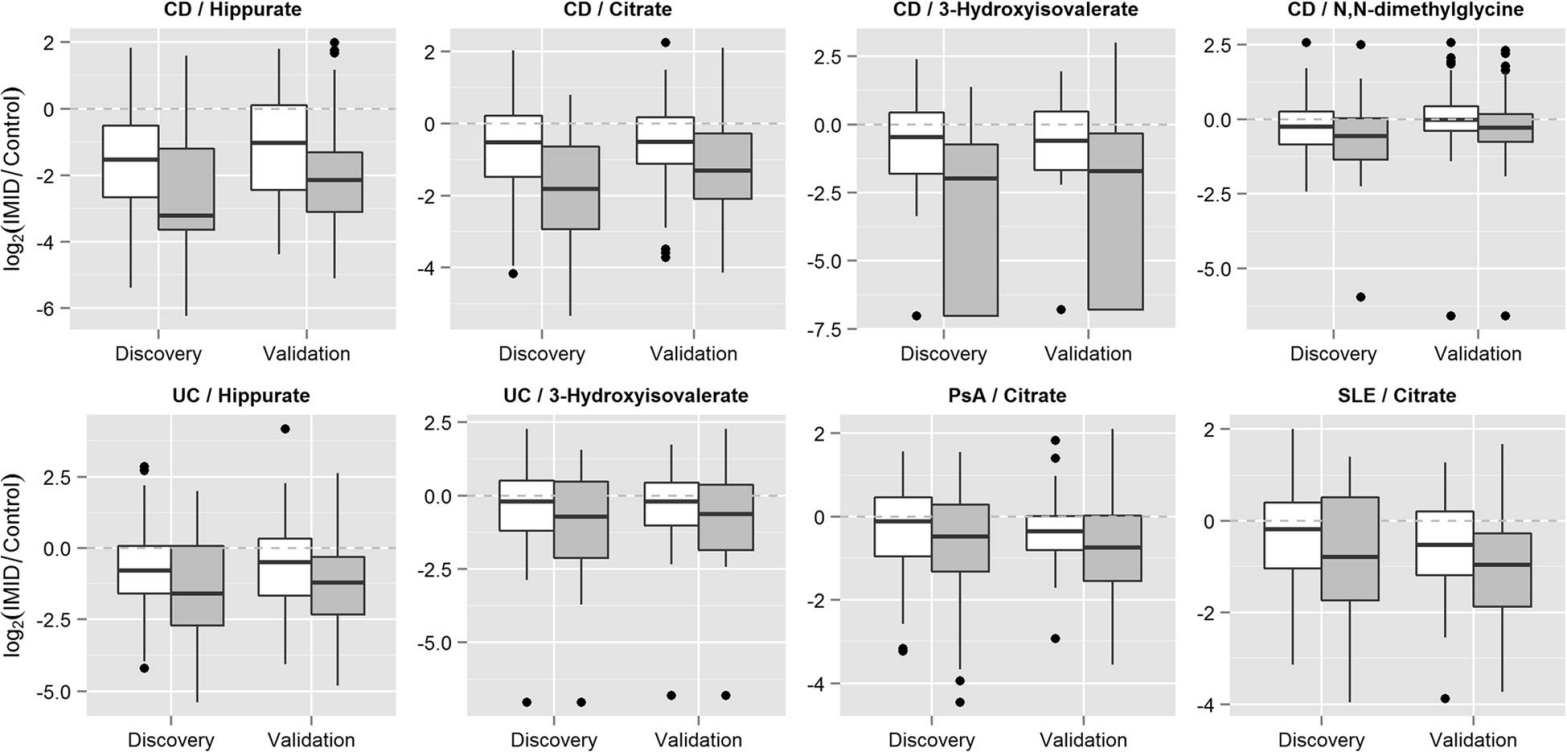
Distribution of metabolite concentrations associated to disease activity. This figure shows the logarithmic concentrations of the metabolites associated to CD disease activity normalized to the median concentration of the control cohort. White and grey bars refer to low and high activity patients, respectively

In the discovery cohort, three metabolites – citrate, hippurate, and 3-hydroxyisovalerate – were found to be significantly associated with disease activity in CD after multiple-test correction (Fig. 4, Additional file 1: Table S4). In particular, CD patients with high levels of disease activity were found to have much lower levels of these three metabolites compared to patients with low disease activity. Using the validation cohort, the association between the low levels of these three metabolites in urine and high disease activity in CD was replicated (*P*_citrate_ = 4.4 × 10^–10^, *P*_hippurate_ = 6.0 × 10^–7^, and *P*_3-hydroxyisovalerate_ = 1.30 × 10^–5^).

After multiple test correction, no other urine metabolite was significantly associated with disease activity. At the nominal level, however, five additional urine metabolites were associated with disease activity in both the discovery and validation cohorts (*P* < 0.05, Additional file 1: Table S4). The direction of the association was the same in both discovery and validation cohorts, which strongly supports the association of these biomarkers as candidates for disease activity monitoring. In UC, high disease activity was associated with low levels of urine hippurate and 3-hydroxyisovaleric acid (*P* = 8.0 × 10^–5^ and *P* = 1.4 × 10^–3^, respectively). In PsA and SLE, patients with higher disease activity had lower levels of citrate (*P* = 1.8 × 10^–5^ and *P* = 1.3 × 10^–3^, respectively). Finally, low levels of N,N-dimethylglycine were also found to be associated with high disease activity in CD (*P* = 9.0 × 10^–4^).

## Discussion

The metabolome represents the collection of small molecules produced by cells and, therefore, its analysis is providing a unique opportunity to identify biological perturbations associated with diseases [29, 45–47]. New technological advances are allowing the characterization of such biochemical variations, revealing unexpected metabolic changes associated with different human pathologies. From a translational perspective, the analysis of the metabolome is beginning to provide new and powerful biomarkers that are highly informative of specific disease processes and, therefore, could lead to more precise and efficient patient management. Despite their prevalence, there remain few studies analyzing the metabolome of IMIDs. In the present study, we report, for the first time, the results of a parallel analysis of the urine metabolome of six of the most prevalent IMIDs – RA, PsA, Ps, SLE, CD, and UC – for the search of clinically relevant biomarkers. Using a two-stage approach we have identified and validated multiple urine metabolites associated with disease diagnosis as well as disease activity. These results provide the most comprehensive analysis of the urine metabolome in IMIDs performed to date, leading to the identification of new biomarker metabolites, as well as providing strong evidence of shared metabolic pathways in this group of diseases.

The present large-scale profiling of the urine metabolome study has found unexpected strong similarities between IMIDs. Some of these metabolite variations were common across all or almost all diseases and, therefore, were considered as hub metabolites. To our knowledge, it is the first time that hub metabolites have been described in IMIDs. Among these metabolites, citrate, a central metabolite of the Krebs oxidative phosphorylation cycle, showed the strongest association to all IMIDs. Despite its essential role in cell energy production, citrate has been recently shown to have important immunologic properties [48], modulating, for example, the production of proinflammatory factors in macrophages or being a critical factor for dendritic cell antigen presentation. Previous studies have found that citrate is present at lower concentrations in the urine of inflammatory bowel disease (IBD) patients compared to controls [49, 50]. In RA and SLE, citrate has also been found to be in lower levels in the serum of patients compared to controls [51, 52]. Here, we show that the previously observed citrate variation in RA and SLE is also detected in urine, a much less invasive sample source than whole blood. Finally, we also demonstrate, for the first time, that Ps and PsA patients also have low concentrations of urine citrate compared to healthy controls. Together, the results of this study provide strong evidence of the presence of hub metabolites that could become “pan-IMID” biomarkers that could be easily measured in routine clinical settings.

The parallel analysis of this group of diseases has led to unique findings. The unsupervised analysis of the urine metabolite associations showed three strong and reproducible clusters of clinically similar IMIDs: (1) IMIDs involving skin affection (i.e., Ps and PsA), (2) inflammatory bowel diseases (i.e., CD and UC), and (3) RA and SLE, two diseases characterized by having a higher prevalence in women. These results correlate with the observed shared genetic risk components observed between different IMIDs using genome-wide association studies [53–56]. For example, CD and UC have shown to share more than 163 disease risk loci [57], Ps an PsA share up to 30 risk loci [58, 59], and SLE and RA have more than 80 common risk variants [60]. To our knowledge, it is the first time that metabolite patterns in urine have shown to etiologically group more similar IMIDs. This result confirms the validity of the urine metabolome in the characterization of biochemical pathways that are specifically associated with this group of diseases.

When assessing the metabolic context of the disease-associated metabolites by integrating the metabolic reactions that link them, the resulting network showed a high degree of overlap of three main metabolic pathways (Fig. 1). From these, the citric acid cycle is the predominant pathway identified, with citrate showing a common association to all the IMIDs. Previous studies have already shown that alterations within this metabolic pathway are related to immunity and inflammation, although the functional implications of the alterations of this pathway are still being investigated [61]. The second major metabolic pathway was the phenylalanine metabolism pathway. The metabolites included in this pathway have shown relevant and specific associations to IBDs in this study. This finding agrees with previous metabolomic studies that have shown the importance of this pathway in the etiology of IBDs [62]. Finally, network analysis also showed an important role for the glycine and serine metabolism pathway in IMIDs. Metabolites within this pathway act as major connectors between the two previous pathways and have been previously related with inflammatory processes. Glycine, the most connected metabolite in the resulting network, has been previously proposed to be an anti-inflammatory and immunomodulatory agent [63]. Although not directly detected by the NMR approach used in this study, our results strongly suggest that glycine could be a highly informative biomarker to the inflammatory processes that characterize IMIDs. Future studies using alternative analysis technologies like mass-spectrometry will help to determine the utility of this metabolite as a clinical biomarker of autoimmune diseases.

In this study, we also demonstrate that the urine metabolome has great potential for assessing disease activity. Citrate, the strongest hub metabolite for IMID diagnosis, was found to correlate with high disease activity in CD, PsA, and SLE. In IBDs, we also demonstrate that hippurate has a very strong correlation with disease activity. Therefore, this urine metabolite could be used not only for early disease diagnosis but also to monitor the level of disease activity in IBDs. This result further strengthens previously reported results that show how changes in the microbiome correlate with the level of inflammation in the gut and disease activity in IBD patients [64–67]. Future studies, aimed at characterizing the interrelation between bacterial species in the gut, tissue inflammation and the urine metabolites identified herein could therefore help to develop more objective and reproducible systems to monitor disease progression in IBDs.

The disease diagnostic models built in this study using the urine metabolites were found to have good performance in all IMIDs. In IBDs in particular, the classifiers were found to predict the disease with very high accuracy. These results are in agreement with previous studies [50, 68, 69] that suggested the use of urine metabolites for the diagnosis of IBDs. Compared to previous studies, we here provide, for the first time, a validation analysis of the diagnostic predictor using an independent and large patient and control cohort. Providing an independent confirmatory analysis is an essential step for any new molecular diagnostic tool [70]. These findings support the analysis of the urine metabolome as a simple, cost-effective and non-invasive approach for the diagnosis of IBDs.

To our knowledge, there is no evidence that the metabolite patterns associated with IMIDs in this study have been previously associated to other diseases. While variations in single metabolites like citrate have been associated with other disease etiologies, the diagnostic ability generated by the combination of multiple metabolites clearly holds a much higher potential to be the approach finally used in the clinical setting. As shown in this study, it is the integration of variation in multiple metabolites that gives the best disease prediction accuracies. In order to further consolidate these diagnostic metabolite patterns as clinically useful tools, the next steps will include the study of the urine metabolome in individuals with pre-diagnostic symptoms as well as longitudinal studies to assess biomarker variability and correlation with specific features of disease progression. Further, future developments of the disease predictors could evaluate the inclusion of other molecular features like the presence of autoantibodies in sera or, even, the identification of additional metabolites in urine using mass-spectrometry approaches. For this latter objective, the results of this study will clearly be a highly valuable starting point.

## Conclusion

We have performed, for the first time, a large-scale high-throughput profiling of the urine metabolome of six of the most prevalent IMIDs. Using a discovery and an independent validation cohort we have identified multiple urine metabolites associated with the diagnosis and the monitoring of disease activity. The parallel evaluation of all six IMIDs has allowed the identification of hub metabolites as well as the characterization of clusters of clinically similar diseases based exclusively on urine metabolite profiles. These common molecular features are in agreement with the shared genetic risk in IMIDs recently identified through genome-wide association studies [54]. Taken together, these results demonstrate the utility of urine metabolomics as a new source for clinically useful biomarkers for this prevalent group of chronic inflammatory diseases.

## Additional file

Additional file 1: Supplementary note (Members of the Immune-Mediated Inflammatory Disease (IMID) Consortium), Supplementary methods, Supplementary Tables (**Table S1:** Sample quality control; **Table S2:** Urine metabolite panel; **Table S3:** List of metabolic associations when comparing phenotypically closer IMID diseases; **Table S4:** List of metabolites with replicated associations to disease activity in Crohn’s disease (CD) patients) and Supplementary Figures (**Figure S1:** Distribution of disease activity indices in the extreme low and high activity patient subgroups; **Figure S2:** Distribution of epidemiological and sample collection variables across the IMID and control groups; **Figure S3:** Distribution of trigonelline concentration according to daily coffee/tea consumption; **Figure S4:** Distribution of trigonelline concentration on each IMID cohort stratified by coffee/tea consumption; **Figure S5:** ROC curves of the diagnostic partial least squares discriminant analysis classification models and metabolite loadings of the CD and UC models). (PDF 2486 kb)

## Abbreviations

AUC: area under the curve
CD: Crohn’s disease
FDR: false discovery rate
IBD: inflammatory bowel disease
IMID: immune-mediated inflammatory disease
N-acetyl AAs: N-acetyl amino acids
NMR: nuclear magnetic resonance
Ps: psoriasis
PsA: psoriatic arthritis
RA: rheumatoid arthritis
ROC: receiver operating characteristic
SLE: systemic lupus erythematosus
UC: ulcerative colitis
