# Supplementary material for: Urine metabolome profiling of immune-mediated inflammatory diseases
Source: BMC Med. 2016 Sep 8;14(1):133. doi: 10.1186/s12916-016-0681-8 (PMC5016926; doi:10.1186/s12916-016-0681-8)

ADDITIONAL INFORMATION

URINE METABOLOME PROFILING IN IMMUNE-MEDIATED INFLAMMATORY DISEASES

Arnald Alonso,[1] Antonio Julià,[1,†] Maria Vinaixa,[2,3] Eugeni Domènech,[4,5] Antonio Fernández-Nebro,[6] Juan D. Cañete, [7] Carlos Ferrándiz,[4] Jesús Tornero,[8] Javier P. Gisbert,[5,9] Pilar Nos,[5,10] Ana Gutiérrez Casbas,[5,11] Lluís Puig,[12] Isidoro González-Álvaro,[9] José A. Pinto-Tasende,[13] Ricardo Blanco,[14] Miguel A. Rodríguez,[2,3] Antoni Beltran,[2,3] Xavier Correig,[2,3] and Sara Marsal,[1,†] on behalf of the IMID Consortium.

[1]Rheumatology Research Group, Vall d'Hebron Hospital Research Institute, Barcelona, Spain. [2]Centre for Omic Sciences, COS-DEEEA-URV-IISPV, Reus, Spain. [3]Metabolomics Platform, CIBERDEM, Reus, Spain. [4]Hospital Universitari Germans Trias i Pujol, Badalona, Spain. [5]CIBERehd, Madrid, Spain. [6]UGC Reumatología, Instituto de Investigación Biomédica (IBIMA), Hospital Regional Universitario de Málaga, Universidad de Málaga, Málaga, Spain. [7]Hospital Clínic de Barcelona and IDIBAPS, Barcelona, Spain. [8]Hospital Universitario Guadalajara, Guadalajara, Spain. [9]Hospital Universitario de la Princesa and IIS-IP, Madrid, Spain. [10]Hospital la Fe, Valencia, Spain. [11]Hospital General de Alicante, Alicante, Spain. [12]Hospital de la Santa Creu i Sant Pau, Barcelona, Spain. [13]Complejo Hospitalario Juan Canalejo, INIBIC, A Coruña, Spain. [14]Hospital Universitario Marqués de Valdecilla, Santander, Spain.

[†]Address correspondence to:
Antonio Julià, PhD, Rheumatology Research Group, Vall d'Hebron Research Institute, Barcelona, Spain. Phone: +34934029082; E-mail: toni.julia@vhir.org.
Sara Marsal, MD, PhD, Rheumatology Research Group, Vall d'Hebron Research Institute, Barcelona, Spain. Phone: +34934029082; Email: sara.marsal@vhir.org.

SUPPLEMENTARY NOTE

The IMID Consortium:

Emilia Fernández[1], Raimon Sanmartí[1], Jordi Gratacós[2], Víctor Manuel Martínez-Taboada[3], Fernando Gomollón[4,5], Esteban Daudén[6], Joan Maymó[7], Rubén Queiró[8], Francisco Javier Lopez Longo[9], Esther Garcia-Planella[10], José Luís Sánchez Carazo[11], Mercedes Alperi-López[8], Carlos Montilla[1], José Javier Pérez-Venegas[12], Benjamín Fernández-Gutiérrez[13], Juan L. Mendoza[13], José Luís López Estebaranz[14], Àlex Olivé[15], Juan Carlos Torre-Alonso[16], Manuel Barreiro-de Acosta[17], David Moreno Ramírez[18], Hèctor Corominas[19], Santiago Muñoz-Fernández[20], José Luis Andreu[21], Fernando Muñoz[22], Pablo de la Cueva[23], Alba Erra[24], Carlos M. González[9], María Ángeles Aguirre-Zamorano[25], Maribel Vera[21], Francisco Vanaclocha[26], Daniel Roig[19], Paloma Vela[27], Cristina Saro[28], Enrique Herrera[29], Pedro Zarco[14], Joan M. Nolla[30], Maria Esteve[31], José Luis Marenco de la Fuente[32], José María Pego-Reigosa[33], Valle García-Sánchez[25], Julián Panés[4,1], Eduardo Fonseca[34], Francisco Blanco[34], Jesús Rodríguez-Moreno[30], Patricia Carreira[26], Julio Ramírez[1], Gabriela Ávila[35], Laia Codó[36], Josep Lluís Gelpí[36], Andrés C. García-Montero[37], Núria Palau[35], María López-Lasanta[35], Raül Tortosa[35]

[1]Hospital Clínic de Barcelona and IDIBAPS, Barcelona. [2]Hospital Parc Taulí, Sabadell. [3]Hospital Universitario Marqués de Valdecilla, Santander. [4]CIBERehd, Madrid. [5]Hospital Clínico Universitario, Zaragoza. [6]Hospital Universitario de la Princesa and IIS-IP, Madrid. [7]Hospital del Mar, Barcelona. [8]Hospital Universitario Central de Asturias, Asturias. [9]Hospital Gregorio Marañón, Madrid. [10]Hospital de la Santa Creu i Sant Pau, Barcelona. [11]Hospital General Universitario, Valencia. [12]Hospital de Jerez de la Frontera, Cádiz. [13]Hospital Clínico San Carlos, IDISSC, Madrid. [14]Hospital Universitario Fundación Alcorcón, Madrid. [15]Hospital Universitari Germans Trias i Pujol, Badalona. [16]Hospital Monte Naranco, Oviedo. [17]Hospital Clínico Universitario, Santiago de Compostela. [18]Hospital Virgen de la Macarena, Sevilla. [19]Hospital Moisès Broggi, Barcelona. [20]Hospital Universitario Infanta Sofía, Madrid. [21]Hospital Universitario Puerta de Hierro, Madrid. [22]Complejo Hospitalario de León, León. [23]Hospital Universitario Infanta Leonor, Madrid. [24]Hospital Sant Rafael, Barcelona. [25]Hospital Universitario Reina Sofía, Instituto Maimónides de Investigación Biomédica de Córdoba (IMIBIC), Universidad de Córdoba, Córdoba. [26]Hospital Universitario Doce de Octubre, Madrid. [27]Hospital General de Alicante, Alicante. [28]Hospital de Cabueñes, Gijón. [29]Hospital Virgen de la Victoria, Málaga. [30]Hospital Universitari de Bellvitge, Barcelona. [31]Hospital Universitari Mútua de Terrassa, Barcelona. [32]Hospital del Valme, Sevilla. [33]Hospital do Meixoeiro, Vigo. [34]Complejo Hospitalario Juan Canalejo, INIBIC, A Coruña. [35]Rheumatology Research Group, Vall d'Hebron Hospital Research Institute, Barcelona. [36]Life Sciences, Barcelona Supercomputing Centre, National Institute of Bioinformatics, Barcelona. [37]Banco Nacional de ADN Carlos III, University of Salamanca, Salamanca. Spain.

SUPPLEMENTARY METHODS

Study subjects. Patient recruitment was performed between June 2007 and December 2010. Patients were recruited from a total of 52 clinical departments from different university hospitals from Spain belonging to the Immune-Mediated Inflammatory Disease Consortium (IMIDC)[1-3]. The IMIDC is a network of clinical and biomedical researchers investigating the molecular basis of immune-mediated inflammatory diseases. Informed consent was obtained from all participants, and protocols were reviewed and approved by local institutional review boards. This study was conducted in accordance with the Declaration of Helsinki principles.

The inclusion and exclusion criteria for each IMID disease were as follows:

- RA: diagnosed according to the revised American College of Rheumatology (ACR) 1987 diagnostic criteria for RA[4], and with >2 years of disease evolution. All patients had joint erosions in either hands or feet. Concomitant cutaneous psoriasis was an exclusion criterion for RA patients.
- PsA: diagnosed according to the CASPAR diagnostic criteria for PsA[5] with >1 year of disease evolution. Exclusion criteria for PA included the presence of any other form of inflammatory arthritis, or rheumatoid factor levels greater than twice the normality threshold.
- CD: diagnosed according to the standard Lennard-Jones diagnostic criteria for CD[6]. Concomitance of any other IMID was an exclusion criterion for CD patients.
- UC: diagnosed according to the Lennard-Jones diagnostic criteria for UC[6]. Concomitance of any other IMID was an exclusion criterion for UC patients.
- SLE: diagnosed according to the ACR diagnostic criteria for SLE[7] and with ≥3 years of disease evolution. Concomitance of any other rheumatic disease, cutaneous psoriasis or inflammatory bowel disease was an exclusion criterion for SLE patients.
- Ps: All eligible PS patients had to have chronic plaque type of PS affecting torso and/or extremities with at least 1 year of duration at the time of recruitment.

All IMID patients were >18 years old at the time of sample collection and were born in Spain. Also, all patients were Caucasian and with all grandparents and parents born in Spain.

Control individuals were recruited in collaboration with the Spanish National DNA Bank from blood bank donors in 13 Spanish hospitals. All the controls were screened for the presence of any autoimmune disorder, as well as for first-degree family occurrence of autoimmune diseases. If positive, the individuals were excluded. All control individuals had also to be Caucasian with all four grandparents born in Spain. Most of the control individuals (i.e. 98%) were ≥40 years old at the time of recruitment.

Study design. The urine metabolomics study in IMIDs was designed following a two-stage approach[8-11]. In the first stage (i.e. discovery stage), we analyzed the urine metabolome of 1,210 IMID patients (203 CD patients, 213 UC patients, 250 RA patients, 169 SLE patients, 190 PsA patients, and 187 Ps patients) and 100 control individuals in order to identify metabolites

associated with disease diagnosis and metabolites associated with disease activity. In the second stage (i.e. validation stage), we used an independent cohort of 1,200 IMID patients (i.e. 200 patients per disease) and 200 control individuals to validate the association of the most significant metabolite markers identified in the discovery stage.

In both the discovery and validation stages, patients within each IMID disease were selected from the IMIDC sample biobank (IMID-Biobank) in order to represent the two extremes of disease activity (i.e. high and low disease activity; Supplementary Figure 1). For each IMID, established scores were used to measure the disease activity at the time of sample collection (Table 1). Within the SLE cohort we used the maximum of the BILAG and SELENA-SLEDAI indices to select the patients according to disease activity. Importantly, patients and controls selection was performed in order to minimize the differences in potential confounding epidemiological variables like gender, age or body mass index, as well as technical variables like fasting time of the individual before sample collection, or the time of the day of sample collection.

Urine sampling. Urine samples from IMID patients were collected in the clinical departments of each of the participating university hospitals. From each patient, 10 mL of urine were obtained and preserved using HCl (1% final concentration). All urine samples were then transported from each center to the IMID-Biobank within 24 hours at room temperature. In the biobank, samples were centrifuged (x600 g, 5 min), aliquoted and immediately stored at -80ºC. Urine samples from the control individuals were also collected using the same procedure and were processed and stored at -80ºC until use in the Spanish national DNA Biobank (BNADN, Salamanca, Spain). For NMR measurement, 200 µL of buffer phosphate (1.5 mM Na_2HPO_4/NaH_2PO_4 in D_2O, pH=7.2) containing 0.62 mM of 3-trimethyl-silyl[2,2,3,3-d4] propionate (TSP) as internal reference were added to 400 µL urine (adjusted pH = 7.2) and the resulting mixture was subsequently transferred to a 5 mm NMR tube.

NMR spectroscopy. ^{1}H-NMR spectra were recorded at 298K on a Bruker Avance III 600 spectrometer operating at a proton frequency of 600.20 MHz using a 5 mm CPTCI triple resonance (^{1}H, ^{13}C, ^{31}P) gradient cryoprobe. One-dimensional ^{1}H pulse experiments were carried out using the nuclear Overhauser effect spectroscopy (NOESY)-presaturation sequence (RD-90º–t_1–90º–t_m–90º ACQ) to suppress the residual water peak. t_1 time was set to 4µs, t_m (mixing time) was 100 ms and recycling delay time was 7 s. The 90° pulse length was calibrated for each sample and varied from 16.3 µs to 18.9 µs. The spectral width was 12.000 Hz (20 ppm), and a total of 64 transients were collected into 64 k data points for each ^{1}H spectrum. The acquired NMR spectra were phased, baseline-corrected and referenced to a TSP signal at δ(0.00 ppm) and used as input to FOCUS processing workflow[12]. Two-dimensional (2D)-^{1}H,^{13}C-HSQC (heteronuclear single quantum correlation) and (2D)-^{1}H-^{1}H COSY (correlation spectroscopy) were acquired in a sample subset for structural confirmation purposes. Several database engines (BBioref AMIX database (Bruker), Chenomx and HMDB[13]) were used for 1D-

resonances assignment. The HMDB[13] and COLMAR ^{1}H,^{13}C-HSQC[14] databases were used for 2D structural confirmation of those 1D assignments.

Spectral processing, metabolite identification, normalization and scaling. In the discovery stage, the raw spectral ^{1}H-NMR profiles were processed using FOCUS software[12]. FOCUS is a complete workflow for NMR data processing that efficiently integrates the different analytical steps, including peak identification, alignment and quantification. One of the main advantages of this method is that it is able to efficiently process large numbers of samples like in the present study. Also, FOCUS includes the RUNAS algorithm, which performs robust peak alignment without the need of a reference spectrum. The RUNAS algorithm has shown to have much better performance than other algorithms, particularly, in metabolomic studies with significantly unaligned spectra.

In this study, spectral processing was performed using spectral windows with a 50% of overlap and a length of 0.077 ppms corresponding to 256 spectral data points. The minimum peak width was set to 0.01 ppms, and the peak frequency threshold was set to 5%. After running FOCUS, we applied several quality control filters at the peak level in order to guarantee the quality of the final set of peaks. First, NMR peaks from windows showing a high degree of sample unalignment were discarded. Significant unalignment was defined when the standard deviation of the shift corrections applied to the spectra was higher than one third of the window length. Second, spectral peaks showing strong evidences of overlapping with other peaks were also discarded.

After applying the quality control filters, we performed the metabolite identification in order to assign to each spectral peak or set of spectral peaks their corresponding metabolite (Table S1). In those cases when multiple spectral peaks represent one same metabolite, we selected the peak showing the highest intensity levels as well as the lowest degree of overlapping (if any). Importantly, exogenous metabolites generated from the metabolism of drugs (e.g. ibuprofen, acetaminophen and 5-aminosalicylic acid) or metabolites related with sample processing (e.g. methanol and ethanol) were identified and subsequently removed from the analysis. Finally, those metabolites that had a high missingness rate (>15% missingness, n=2), were also discarded from the analysis.

In the validation stage, the spectral ^{1}H-NMR profiles from the case and control urine samples were also processed using FOCUS software. In this case, and in order to improve the accuracy of the data analysis, the NMR signal processing was restricted to the metabolites identified in the discovery stage. Consequently, the parameters used in FOCUS NMR signal processing were set to accurately analyze the spectral regions that were informative in the discovery stage.

Intensity normalization is an important step in ^{1}H-NMR spectral processing and it can be critical when analyzing urine samples[9,15-17]. The objective of this analytical step is to correct the variability introduced by the dilution of metabolites naturally present in urine samples. Normalization to creatinine area was discarded, since the urine concentrations of this metabolite

have been associated to gender, muscle mass or dietary habits[9,18]. We therefore chose normalization to the total spectral area (i.e. area under the curve; AUC), a robust method that has been extensively used in multiple urine metabolomics studies[19-22]. In order to avoid the bias introduced by metabolites present in high concentrations in urine, we excluded the following regions from the total spectral calculation: creatinine (4.00 to 4.10 and 3.00 to 3.10 ppm), water (4.70 to 5.00 ppm), citrate (from 2.50 to 2.71), hippurate (from 7.80 to 7.86), eretic (10.50 to 11.50 ppm), and TSP (-0.50 to 0.50 ppm). Nonetheless, when comparing the AUC normalization values to the creatinine normalization values in both the discovery and validation cohorts, we found relatively high levels of correlation between the two normalization methods (r=0.87 and r=0.77, respectively). In order to avoid the biases introduced by outlying metabolite values in the subsequent multivariate association analyses, we applied a logarithmic transformation to the metabolite quantifications[23]. In order to avoid infinite transformed values generated from the transformation of zero values, we substitute them by 0.1 times the minimum non-zero value.

Sample quality control. Sample quality control was performed in two steps. First, we used the AUC normalization values and the missingness rates of the final metabolite panel to identify outlier samples. Samples showing AUC normalization values lower than 1st percentile or higher than 99th percentile were removed. We also removed samples with missingness rates higher than 20%. Second, we performed a principal component analysis (PCA) on the transformed intensities of the final metabolite panel. Before applying the PCA, peak intensities were scaled using Pareto scaling method as described previously[24]. Once scaled, we iteratively applied the PCA to compute the principal component scores of all samples. At each iteration, outlier samples (defined as samples having PC scores >4 times the standard deviation of the first and second principal components) were excluded. This process was repeated until no outlier samples were left.

Epidemiological and sample collection variables. There is substantial evidence that metabolite levels in human body fluids are influenced by many different factors that can act as confounders in disease association analyses[8,10,11,25]. In order to control for these factors, we collected information on several potentially confounding variables. This potential confounders included epidemiological variables (i.e. gender, age, and body mass index), variables associated with the urine collection process (i.e. collection time and fasting time), and variables summarizing lifestyle and dietary habits. With regards to lifestyle, we specifically analyzed three lifestyle variables which are smoking status, amount of leisure time physical activity and physical labor at work. Variables of dietary habits included: (a) weekly consumption of meat, fish, eggs, dietary products, vegetables, legumes, cereals, and sweets, and (b), daily consumption of coffee/tea, beer and wine.

Statistical analysis. The association analyses between each metabolite and the phenotype of interest were performed using linear regression analysis. In order to discard the effect of potential confounders in each metabolite association, we included the potential confounders of

the metabolite being tested in the linear regression model as described previously[26]. This model corresponds to the following formula:

$$C_i \sim Ph + X_1^i + \cdots + X_N^i$$

where C_i refers to the concentrations of metabolite *i*, *Ph* refers to the phenotypic variable being tested and $X_1^i, \ldots X_N^i$ refers to the set of N potential confounders for metabolite *i*. Potential confounders were defined as those epidemiological or technical variables showing a nominal association with the metabolite concentrations in the control cohort (univariate linear regression, P<0.05). Including these covariates when adjusting the multivariate linear regression model we efficiently controlled their potential confounding effect over the metabolite association with each disease and/or disease activity.

Previous to clinical association analyses we verified that drug therapies were not associated with any metabolite in all the evaluated IMID diseases. In this analysis we used the information of the patients of each IMID that were under each considered treatment at the urine sample collection time. We included in the analysis the most established therapy groups for IMIDs: biological therapies (i.e. etanercept, infliximab and adalimumab), immunomodulators (methotrexate, leflunomide, azathioprine and salazopyrin), anti-inflammatory drugs (5-aminosalicylic acid, ibuprofen and diclofenac) and glucocorticosteroids (prednisone and deflazacort). No relevant or significant associations were found.

Three types of clinical association analyses of urine metabolites were performed:

- Diagnostic biomarkers: the urine metabolite levels of each IMID disease were compared against the levels in the control cohort. In this analysis approach we used all the patients from each IMID and we also performed an analysis using only the patients showing high disease activity levels.
- Differential diagnostic biomarkers: the metabolite levels were compared between IMID diseases that have the most similar clinical features (i.e. CD vs. UC, RA vs. PsA, Ps vs. PsA, and RA vs. SLE).
- Activity biomarkers: within each IMID disease, we compared the urine metabolite levels between high and low disease activity groups of patients.

Correction for multiple testing was performed using the false discovery rate (FDR) method[27]. After multiple test correction, a significant metabolite association (i.e. FDR-adjusted *P*-Value <0.05) was subsequently selected for replication in the independent validation dataset. In the validation analysis, multiple test correction was also performed using the FDR approach. Combined *P*-Values of the discovery and validation datasets were computed using Fisher's method[28].

IMID diseases and metabolites were clustered according to the replicated metabolite-disease associations using hierarchical clustering (i.e. function “heatmap.2” of the R package “Gplots” [29]). The input data for the clustering was a matrix (metabolites x disease), containing the association $–\log_{10}$(*P*-Values) between the corresponding row metabolite and the corresponding column disease.

The receiver operating characteristic (ROC) curve analysis for metabolomic diagnostic biomarkers was performed to evaluate the prediction power of the urine metabolome to distinguish between healthy subjects and IMID patients as described previously[23,30]. The area under de curve (AUC) metric was used to assess the performance of the classifiers. The ROC curves were computed using the “pROC” R package[31]. The optimal threshold was determined by maximizing the sum of the sensitivity and specificity parameters across de ROC curve. The prediction models were built using only those metabolites associated in the discovery cohort for each IMID disease. The concentrations of each cohort (i.e. discovery and validation) were scaled using Pareto scaling based on the mean and variance of the corresponding control cohorts. The classification models were based on the PLS-DA algorithm included in the “mixOmics” R package[32]. In this analysis, two latent variables and the entire set of metabolites associated at the single level in the discovery cohort were used. We used the validation dataset to assess the performance of the PLS-DA model built with the entire discovery dataset. This approach corresponds to a true validation on a completely independent dataset.

SUPPLEMENTARY REFERENCES

1 Julià, A. *et al.* A genome-wide association study identifies a novel locus at 6q22.1 associated with ulcerative colitis. *Human Molecular Genetics* **23**, 6927-6934 (2014).

2 Julià, A. *et al.* A genome-wide association study on a southern European population identifies a new Crohn's disease susceptibility locus at RBX1-EP300. *Gut* **62**, 1440-1445 (2013).

3 Julià, A. *et al.* Risk variants for psoriasis vulgaris in a large case–control collection and association with clinical subphenotypes. *Human Molecular Genetics* **21**, 4549-4557 (2012).

4 Arnett, F. C. *et al.* The american rheumatism association 1987 revised criteria for the classification of rheumatoid arthritis. *Arthritis & Rheumatism* **31**, 315-324, doi:10.1002/art.1780310302 (1988).

5 Taylor, W. *et al.* Classification criteria for psoriatic arthritis: Development of new criteria from a large international study. *Arthritis & Rheumatism* **54**, 2665-2673, doi:10.1002/art.21972 (2006).

6 Lennard-Jones, J. E. Classification of Inflammatory Bowel Disease. *Scandinavian Journal of Gastroenterology* **24**, 2-6, doi:10.3109/00365528909091339 (1989).

7 Tan, E. M. *et al.* The 1982 revised criteria for the classification of systemic lupus erythematosus. *Arthritis & Rheumatism* **25**, 1271-1277, doi:10.1002/art.1780251101 (1982).

8 Psihogios, N. G., Gazi, I. F., Elisaf, M. S., Seferiadis, K. I. & Bairaktari, E. T. Gender-related and age-related urinalysis of healthy subjects by NMR-based metabonomics. *NMR in Biomedicine* **21**, 195-207, doi:10.1002/nbm.1176 (2008).

9 Rasmussen, L. *et al.* Standardization of factors that influence human urine metabolomics. *Metabolomics* **7**, 71-83, doi:10.1007/s11306-010-0234-7 (2011).

10 Slupsky, C. M. *et al.* Investigations of the Effects of Gender, Diurnal Variation, and Age in Human Urinary Metabolomic Profiles. *Analytical Chemistry* **79**, 6995-7004, doi:10.1021/ac0708588 (2007).

11 Walsh, M. C., Brennan, L., Malthouse, J. P. G., Roche, H. M. & Gibney, M. J. Effect of acute dietary standardization on the urinary, plasma, and salivary metabolomic profiles of healthy humans. *The American Journal of Clinical Nutrition* **84**, 531-539 (2006).

12 Alonso, A. *et al.* Focus: A Robust Workflow for One-Dimensional NMR Spectral Analysis. *Analytical Chemistry* **86**, 1160-1169, doi:10.1021/ac403110u (2013).

13 Wishart, D. S. *et al.* HMDB 3.0—The Human Metabolome Database in 2013. *Nucleic Acids Research* **41**, D801-D807 (2013).

14 Bingol, K. *et al.* Unified and Isomer-Specific NMR Metabolomics Database for the Accurate Analysis of 13C–1H HSQC Spectra. *ACS Chemical Biology* **10**, 452-459, doi:10.1021/cb5006382 (2015).

15 Craig, A., Cloarec, O., Holmes, E., Nicholson, J. K. & Lindon, J. C. Scaling and Normalization Effects in NMR Spectroscopic Metabonomic Data Sets. *Analytical Chemistry* **78**, 2262-2267, doi:10.1021/ac0519312 (2006).

16 Kohl, S. *et al.* State-of-the art data normalization methods improve NMR-based metabolomic analysis. *Metabolomics* **8**, 146-160, doi:10.1007/s11306-011-0350-z (2012).

17 Weljie, A. M., Newton, J., Mercier, P., Carlson, E. & Slupsky, C. M. Targeted Profiling: Quantitative Analysis of 1H NMR Metabolomics Data. *Analytical Chemistry* **78**, 4430-4442, doi:10.1021/ac060209g (2006).

18 Stella, C. *et al.* Susceptibility of Human Metabolic Phenotypes to Dietary Modulation. *Journal of Proteome Research* **5**, 2780-2788, doi:10.1021/pr060265y (2006).

19 Kapoor, S. R. *et al.* Metabolic profiling predicts response to anti-tumor necrosis factor alpha therapy in patients with rheumatoid arthritis. *Arthritis Rheum.* **65**, 1448-1456. doi: 1410.1002/art.37921. (2013).

20 Schicho, R. *et al.* Quantitative Metabolomic Profiling of Serum, Plasma, and Urine by 1H NMR Spectroscopy Discriminates between Patients with Inflammatory Bowel Disease and Healthy Individuals. *Journal of Proteome Research* **11**, 3344-3357, doi:10.1021/pr300139q (2012).

21 Williams, H. R. T. *et al.* Characterization of Inflammatory Bowel Disease With Urinary Metabolic Profiling. *Am J Gastroenterol* **104**, 1435-1444, doi:http://www.nature.com/ajg/journal/v104/n6/suppinfo/ajg2009175s1.html (2009).

22 Young, S. P. *et al.* The impact of inflammation on metabolomic profiles in patients with arthritis. *Arthritis Rheum.* **65**, 2015-2023. doi: 2010.1002/art.38021. (2013).
23 Xia, J., Broadhurst, D., Wilson, M. & Wishart, D. Translational biomarker discovery in clinical metabolomics: an introductory tutorial. *Metabolomics* **9**, 280-299, doi:10.1007/s11306-012-0482-9 (2013).
24 van den Berg, R., Hoefsloot, H., Westerhuis, J., Smilde, A. & van der Werf, M. Centering, scaling, and transformations: improving the biological information content of metabolomics data. *BMC Genomics* **7**, 142 (2006).
25 Enea, C. *et al.* 1H NMR-based metabolomics approach for exploring urinary metabolome modifications after acute and chronic physical exercise. *Anal Bioanal Chem* **396**, 1167-1176, doi:10.1007/s00216-009-3289-4 (2010).
26 Shin, S.-Y. *et al.* An atlas of genetic influences on human blood metabolites. *Nat Genet* **46**, 543-550, doi:10.1038/ng.2982

http://www.nature.com/ng/journal/v46/n6/abs/ng.2982.html#supplementary-information (2014).
27 Benjamini, Y. & Hochberg, Y. Controlling the False Discovery Rate: A Practical and Powerful Approach to Multiple Testing. *Journal of the Royal Statistical Society. Series B (Methodological)* **57**, 289-300, doi:10.2307/2346101 (1995).
28 Fisher, R. A. *Statistical Methods for Research Workers. 5th ed*. (Edinburgh, 1934).
29 Warnes, G. R. *et al.* gplots: Various R programming tools for plotting data. *R package version* **2** (2009).
30 Alonso, A., Marsal, S. & Julià, A. Analytical methods in untargeted metabolomics: state of the art in 2015. *Frontiers in Bioengineering and Biotechnology* **3** (2015).
31 Robin, X. *et al.* pROC: an open-source package for R and S+ to analyze and compare ROC curves. *BMC Bioinformatics* **12**, 77 (2011).
32 González, I., Lê Cao, K. & Déjean, S. MixOmics: Omics data integration project. *R package version* **3** (2011).

SUPPLEMENTARY TABLES

Supplementary Table 1. Sample quality control. Number of samples passing each one of the three quality control stages.

QC stage	Discovery		Validation	
	Controls	IMIDs	Controls	IMIDs
Initial	100	1210	200	1200
Sample quality	95	1210	199	1198
Missingness >20%	94	1183	196	1156
PCA	93	1180	196	1152

Supplementary Table 2. Urine metabolite panel. The list of metabolites identified in urine that pass all the quality control filters and that were included in the subsequent statistical association analyses.

Metabolite	δ(^{1}H)[ppm]	δ(^{13}C)[ppm]	Moiety	QC_{IP}†	HMDB
Trigonelline*	s-9.13	-	C3-N+	0.10	HMDB00875
Hippurate*	d- 7.85	129.9	C2-ring	0.80	HMDB00714
Phenylacetylglycine*	m-7.43	131.01	C3,C5 aromatic	0.70	HMDB00821
Unknown 1	s-7.25	-		0.10	-
Unknown 2	s-7.21			0.50	-
Tyrosine*	d-7.15	133.4	C2,6-ring	0.25	HMDB00158
Creatinine*	s -4.05	59.2	CH_2-N	0.95	HMDB00562
Unknown 3	s-4.03	58.5		0.95	-
Phosphocreatine	s-3.94	56.4	-CH_2-	0.90	HMDB01511
Unknown 4	s-3.91			0.85	-
Glycine*	s -3.56	44.1	aCH_2	0.95	HMDB00123
Phenylacetate*	s-3.50	-	aCH_2	0.35	HMDB00209
TMAO*	s -3.29	62.3	$(CH_3)_3$-N	0.95	HMDB00925
Carnitine*	s- 3.23	-	$(CH_3)_3$-N	0.75	HMDB00062
o-phosphocholine*	s- 3.20	56.4	$(CH_3)_3$-N	0.75	HMDB01565
Dimethylsulfone*	s- 3.12	44	2×(CH_3)	0.70	HMDB04983
Unknown 5	s-3.11			0.60	-
N,N-dimethylglycine*	s-2.93	46.3	2×(CH_3)	0.55	HMDB00092
Unknown 6	s-2.78			0.30	-
N,N-dimethylamine*	s- 2.72	37.5	$(CH_3)_2$-NH	0.90	HMDB00087
Citrate*	s- 2.69	48.5	half-CH_2	0.90	HMDB00094
3-Hydroxyisovalerate*	s- 2.35	-	aCH_2	0.75	HMDB00754
Aminoadipate*	t-2.27	34.2	dCH_2	0.60	HMDB00510
Acetoacetate*	d-2.22	-	CH_3-	0.45	HMDB00060
Unknown 7	s-2.17	25.4	CH3 without coupling	0.70	-
N-acetyl Aas*	s-2.07	-	CH_3-	0.80	-
Unknown 8	s-2.05			0.80	-
Free acetate*	s-1.91	25.9	CH_3-	0.70	HMDB00042
Thymine	s-1.86	-	CH_3-	0.40	HMDB00262
Alanine*	d-1.48	19	CH_3-	0.50	HMDB00161
Lactate*	d-1.33	22.9	CH_3-	0.55	HMDB00190
Methylsuccinate*	d-1.07	19.7	aCH_3	0.35	HMDB01844
Butyrate*	t-0.89	-	CH_3-	0.40	HMDB00039

* Metabolites identified in urine by previous ^{1}H-NMR studies.

† Mean intensity percentile of the peak maximums considering all the spectral data points.

Supplementary Table 3. List of metabolic associations when comparing phenotypically closer IMID diseases. This table shows the replicated associations between metabolite concentrations and IMID diseases pairs.

Metabolite	$IMID_1$	$IMID_2$	$\log_2(IMID_2/IMID_1)_{DISC}$	P-$Value_{DISC}$	$\log_2(IMID_2/IMID_1)_{DISC}$	P-$Value_{VAL}$	P-$Value_{COMB}$
Unknown 7	CD	UC	3.090 (2.384,3.795)	2.63E-12	2.552 (1.791,3.313)	5.78E-08	6.70E-18
Citrate	CD	UC	0.604 (0.401,0.807)	1.41E-06	0.423 (0.223,0.622)	5.28E-04	1.60E-08
Hippurate	CD	UC	0.730 (0.458,1.001)	1.25E-05	0.558 (0.302,0.814)	3.65E-04	9.20E-08
Unknown 7*	RA	PsA	-1.240 (-1.787,-0.693)	2.12E-04	-0.787 (-1.378,-0.196)	2.87E-02	7.90E-05
Tyrosine*	RA	PsA	-1.047 (-1.555,-0.539)	7.45E-04	-0.597 (-1.018,-0.177)	1.97E-02	1.80E-04
Phenylacetate†	RA	SLE	-0.240 (-0.383,-0.097)	5.95E-03	-0.317 (-0.482,-0.152)	1.71E-03	1.30E-04
3-Hydroxyisovalerate†	CD	UC	0.550 (0.163,0.937)	1.97E-02	0.920 (0.470,1.370)	8.23E-04	2.00E-04

* Validated at the nominal level

† Associated at the nominal level in both the discovery and validation cohorts

Supplementary Table 4. List of metabolites with replicated associations to disease activity in CD patients. This table shows the activity association results that have been replicated.

IMID / Metabolite	$log_2(IMID_{HIGH}/IMID_{LOW})_{DISC}$	*P*-Value$_{DISC}$	$log_2(IMID_{HIGH}/IMID_{LOW})_{VAL}$	*P*-Value$_{VAL}$	*P*-Value$_{COMB}$
CD/Citrate	-1.225 (-1.594,-0.857)	1.20E-07	-0.660 (-0.941,-0.380)	1.40E-04	4.40E-10
CD/Hippurate	-1.057 (-1.482,-0.631)	6.00E-05	-0.803 (-1.180,-0.425)	5.50E-04	6.00E-07
CD/3-Hydroxyisovalerate	-1.761 (-2.455,-1.066)	4.30E-05	-0.973 (-1.665,-0.281)	2.10E-02	1.30E-05
PsA/Citrate*	-0.494 (-0.772,-0.217)	3.70E-03	-0.537 (-0.780,-0.294)	3.50E-04	1.80E-05
UC/Hippurate*	-0.636 (-0.988,-0.284)	3.20E-03	-0.643 (-0.981,-0.305)	1.90E-03	8.00E-05
CD/N,N-dimethylglycine*	-0.475 (-0.754,-0.196)	5.40E-03	-0.445 (-0.748,-0.142)	1.60E-02	9.00E-04
SLE/Citrate*	-0.463 (-0.804,-0.122)	2.60E-02	-0.582 (-0.919,-0.245)	5.00E-03	1.30E-03
UC/3-Hydroxyisovalerate*	-0.765 (-1.259,-0.271)	1.10E-02	-0.851 (-1.412,-0.291)	1.30E-02	1.40E-03

* Nominal level of association

SUPPLEMENTARY FIGURES

Supplementary Figure 1. Distribution of disease activity indices in the extreme low and high activity patient subgroups. This figure shows the distribution of the disease activity indices in the low (L) and high (H) activity subgroups of each IMID disease.

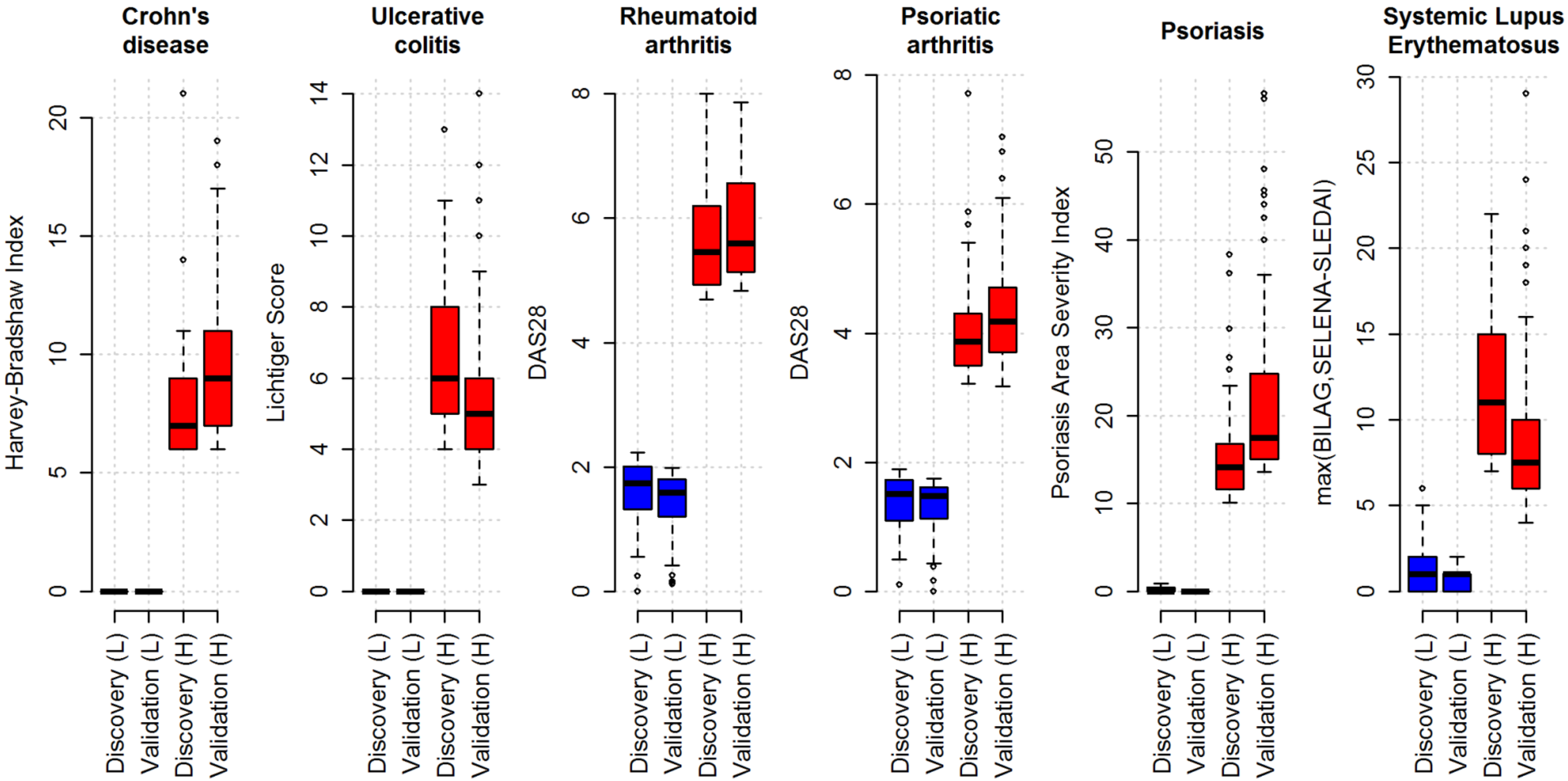

Supplementary Figure 2. Distribution of epidemiological and sample collection variables across the IMID and control groups. Distribution of gender, current smoking behaviour, fast time before sample collection, sample collection time, age at collection time and body mass index across the studied patient and control cohorts in the discovery and validation datasets.

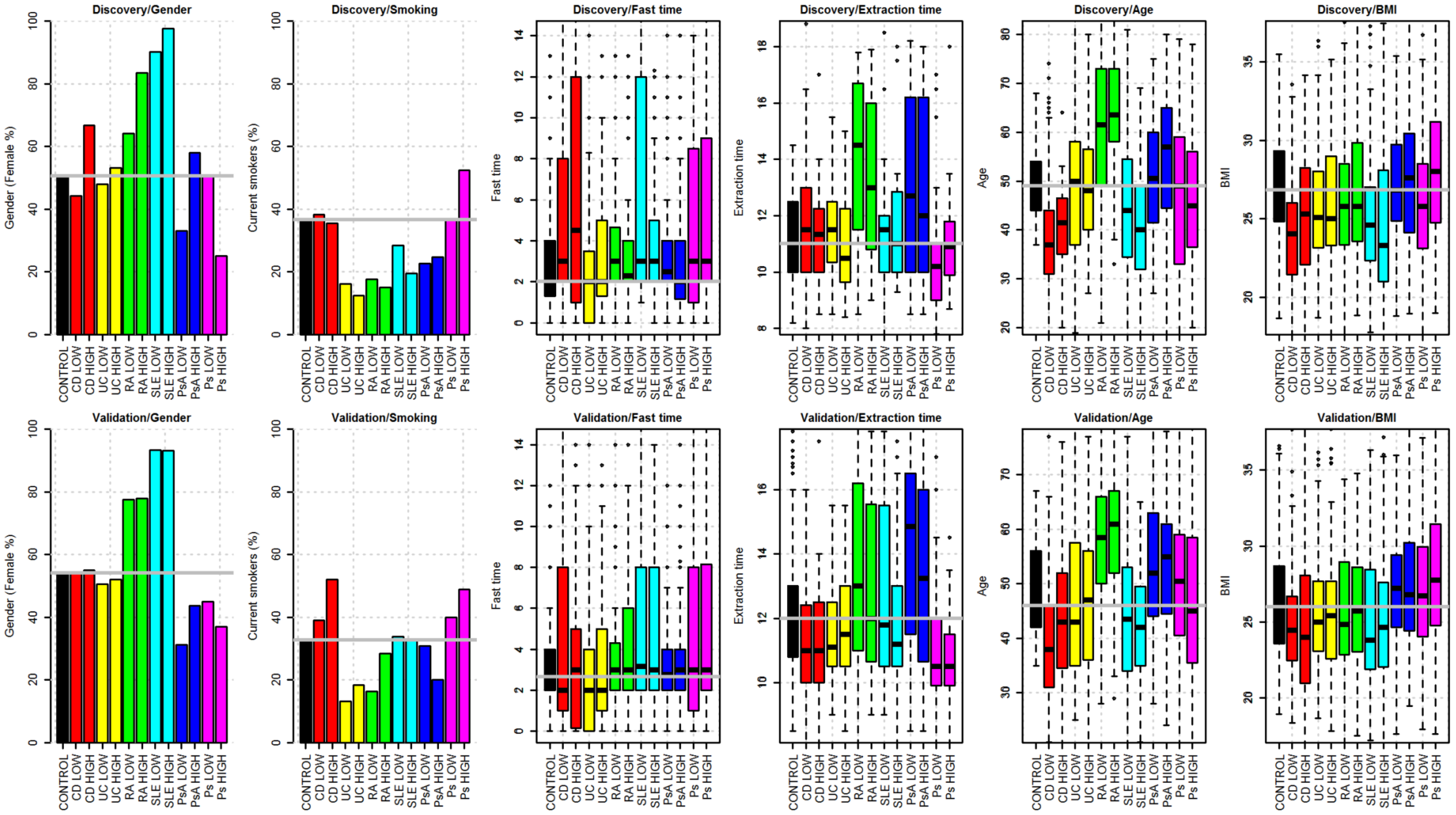

Supplementary Figure 3. Distribution of trigonelline concentration according to daily coffee/tea consumption. This figure shows the distribution of logarithmic trigonelline levels in the control cohort depending on the reported daily consumption of coffee/tea cups.

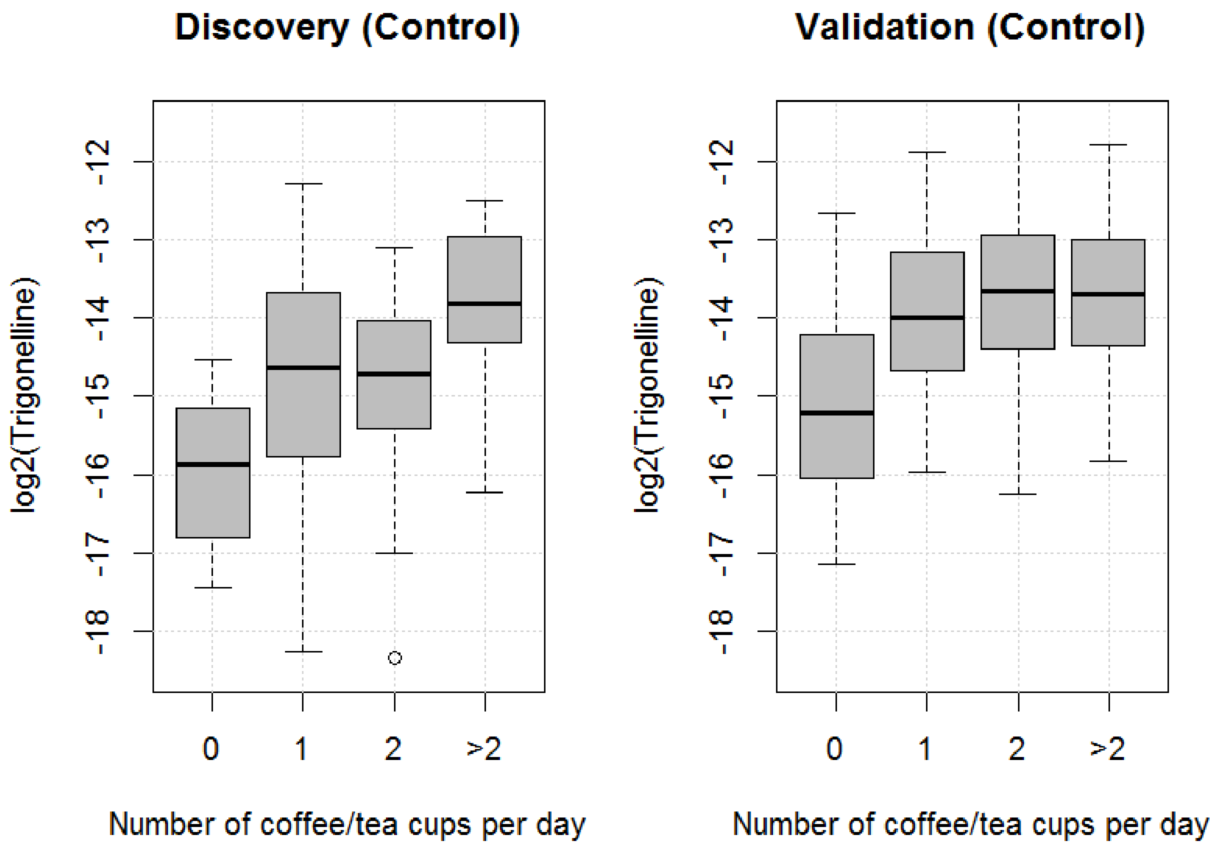

Supplementary Figure 4. Distribution of trigonelline concentration on each IMID cohort stratified by coffee/tea consumption. This figure shows the distribution of normalized trigonelline levels on each IMID cohort. (A) refers to the complete set of patients while (B) and (C) respectively refers to patients having 0 or 1 coffee/tea cups per day.

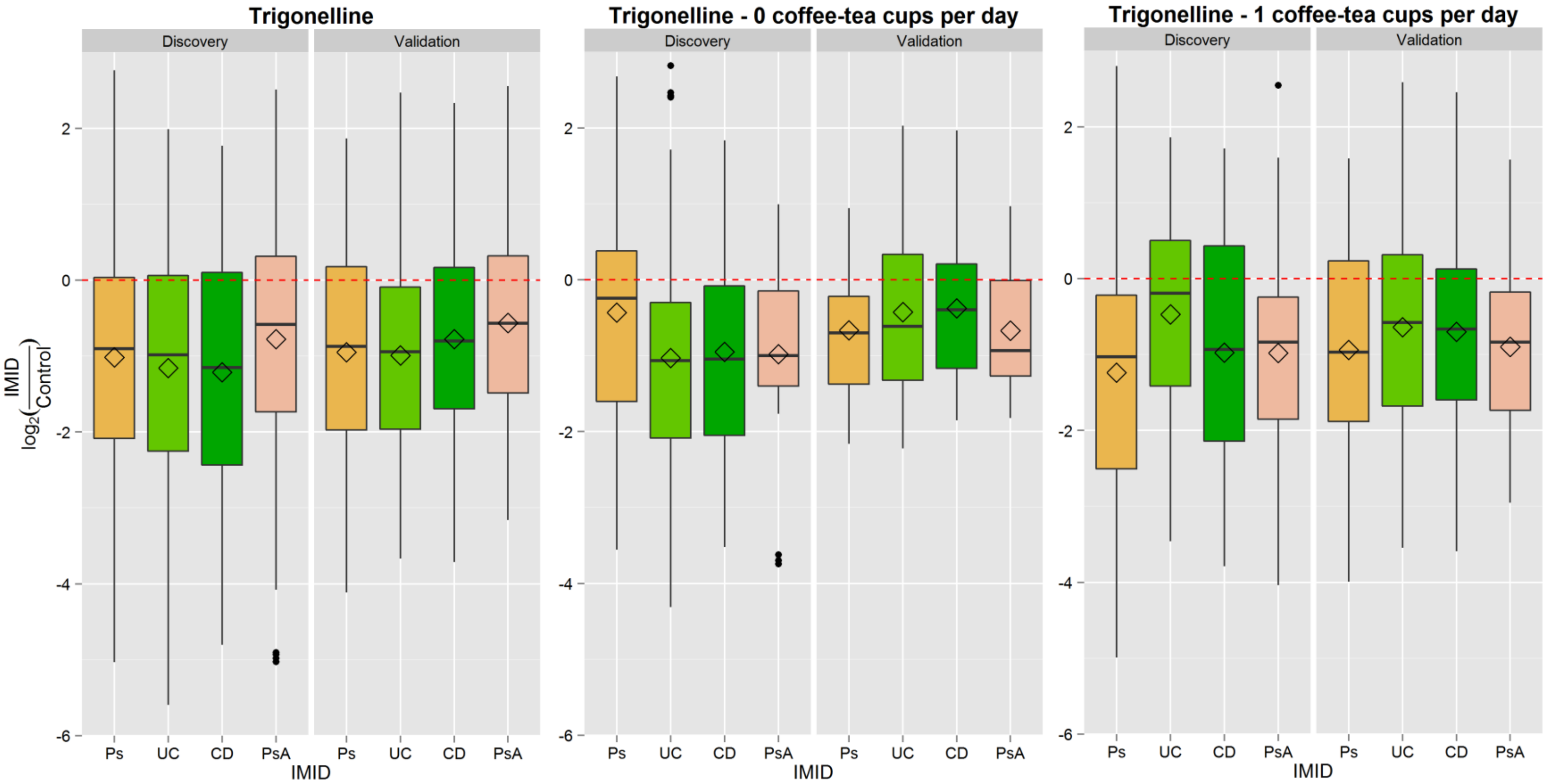

Supplementary Figure 5. ROC curves of the diagnostic PLS-DA classification models and metabolite loadings of the CD and UC models. (A) ROC curves evaluated in the discovery and validation datasets for the classification models build using the discovery dataset. (B) Loadings of each metabolite included in the PLS-DA classification models for UC and CD.

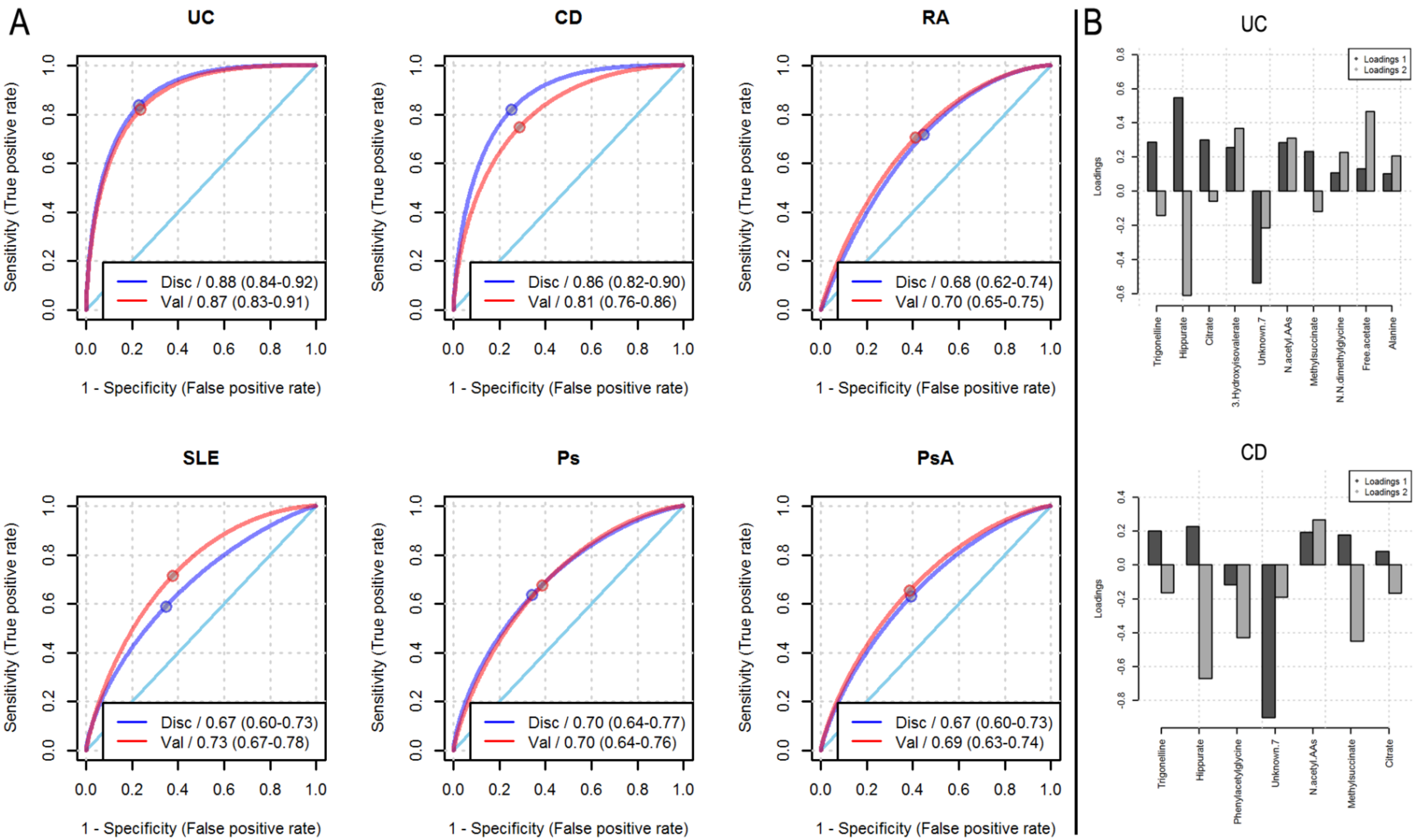

Supplement: Additional file 1: — Supplementary note (Members of the Immune-Mediated Inflammatory Disease (IMID) Consortium), Supplementary methods, Supplementary Tables (Table S1: Sample quality control; Table S2: Urine metabolite panel; Table S3: List of metabolic associations when comparing phenotypically closer IMID diseases; Table S4: List of metabolites with replicated associations to disease activity in Crohn’s disease (CD) patients) and Supplementary Figures (Figure S1: Distribution of disease activity indices in the extreme low and high activity patient subgroups; Figure S2: Distribution of epidemiological and sample collection variables across the IMID and control groups; Figure S3: Distribution of trigonelline concentration according to daily coffee/tea consumption; Figure S4: Distribution of trigonelline concentration on each IMID cohort stratified by coffee/tea consumption; Figure S5: ROC curves of the diagnostic partial least squares discriminant analysis classification models and metabolite loadings of the CD and UC models). (PDF 2486 kb) [file 12916_2016_681_MOESM1_ESM.pdf]
